# Distribution of delirium motor subtypes in the intensive care unit: a systematic scoping review

**DOI:** 10.1186/s13054-022-03931-3

**Published:** 2022-03-03

**Authors:** Kirstine N. la Cour, Nina C. Andersen-Ranberg, Sarah Weihe, Lone M. Poulsen, Camilla B. Mortensen, Cilia K. W. Kjer, Marie O. Collet, Stine Estrup, Ole Mathiesen

**Affiliations:** 1 Department of Anesthesiology, Centre for Anaesthesiological Research, Zealand University Hospital, Koege, Denmark; 2 Department of Intensive Care, Copenhagen University Hospital, Rigshospitalet, Denmark; 3 Department of Clinical Medicine, Copenhagen University, Copenhagen, Denmark

**Keywords:** Critically ill, Delirium, Delirium motor subtype, Intensive care

## Abstract

**Background:**

Delirium is the most common cerebral dysfunction in the intensive care unit (ICU) and can be subdivided into a hypoactive, hyperactive, or mixed motor subtype based on the clinical manifestation. The aim of this review was to describe the distribution, pharmacological interventions, and outcomes of delirium motor subtypes in ICU patients.

**Methods:**

This systematic scoping review was performed according to the PRISMA-ScR and Cochrane guidelines. We performed a systematic search in six major databases to identify relevant studies. A meta-regression analysis was performed where pooled estimates with 95% confidence intervals were computed by a random effect model.

**Results:**

We included 131 studies comprising 13,902 delirious patients. There was a large between-study heterogeneity among studies, including differences in study design, setting, population, and outcome reporting. Hypoactive delirium was the most prevalent delirium motor subtype (50.3% [95% CI 46.0–54.7]), followed by mixed delirium (27.7% [95% CI 24.1–31.3]) and hyperactive delirium (22.7% [95% CI 19.0–26.5]). When comparing the delirium motor subtypes, patients with mixed delirium experienced the longest delirium duration, ICU and hospital length of stay, the highest ICU and hospital mortality, and more frequently received administration of specific agents (antipsychotics, α2-agonists, benzodiazepines, and propofol) during ICU stay. In studies with high average age for delirious patients (> 65 years), patients were more likely to experience hypoactive delirium.

**Conclusions:**

Hypoactive delirium was the most prevalent motor subtype in critically ill patients. Mixed delirium had the worst outcomes in terms of delirium duration, length of stay, and mortality, and received more pharmacological interventions compared to other delirium motor subtypes. Few studies contributed to secondary outcomes; hence, these results should be interpreted with care. The large between-study heterogeneity suggests that a more standardized methodology in delirium research is warranted.

**Supplementary Information:**

The online version contains supplementary material available at 10.1186/s13054-022-03931-3.

## Introduction

Delirium is a cerebral dysfunction affecting nearly one-third of patients in the intensive care unit (ICU) [1, 2]. The clinical manifestations of delirium exhibit great variance. Lipowski et al. [3] were the first to suggest the use of hyperactive and hypoactive motor subtypes to describe the different manifestations, and with the term mixed delirium being introduced shortly after [4].

A patient with *hyperactive delirium* is in an agitated state of mind and may display symptoms of aggression and restlessness, whereas a patient with *hypoactive delirium* is in an apathetic state of mind and may display symptoms of lethargy, depression, and stupor. In *mixed delirium*, the patient shifts between hyperactive and hypoactive delirium. All delirium motor subtypes experience elements of the hallmark symptoms in delirium such as confusion and inattention [5]. Delirium may also be divided into other clinical phenotypes based on underlying mechanisms, e.g., sedation- or hypoxic-associated delirium [6].

We still have limited knowledge about the pathophysiology of delirium. The *Neurotransmitter Hypothesis* suggests that specific neurotransmitter interactions with the cholinergic pathway might determine delirium motor subtype [7–9]. Currently no evidence-based pharmacological treatment exists for this severe condition [1, 10]. Delirium in the ICU is associated with increased short- and long-term impairments [2, 11–13]. The extent of impairments and whether they are associated with delirium motor subtypes are still to be determined. Recently, hypoactive delirium was associated with the poorest prognosis of survival compared with mixed and hyperactive delirium [14, 15], whereas another study found that the duration of the different motoric subtypes was not associated with long-term functional outcomes [16]. Considering the heterogeneity in etiology, manifestation, treatment response, and outcomes of the three delirium motor subtypes, it has been speculated that these in essence represent different disease entities [17, 18].

A key to future treatment strategies may depend on increased knowledge of distribution and current treatments of the delirium motor subtypes. In this scoping review, we therefore aimed to describe distribution, pharmacological interventions, and outcomes associated with delirium motor subtypes in adult critically ill patients.

## Methods

This review was conducted in accordance with Preferred Reporting Items for Systematic reviews and Meta-Analyses extension for Scoping Reviews (PRISMA-ScR) [19] and preregistered the 26th of September 2017 at PROSPERO (ID:CRD42017076503). The full protocol is available from the corresponding author on request.

### Literature search

The literature search strategy was designed in collaboration with a Health Sciences Librarian with expertise in systematic reviews and was performed twice, with the last search in April 2021 in the following databases: Cochrane Central Register of Controlled Trials (CENTRAL), MEDLINE/PubMed, Ovid/EMBASE, Cumulative Index to Nursing & Allied Health Literature (CINAHL), PsycINFO and Google Scholar. We manually screened ClinicalTrials.gov, EU Clinical Trial Registry, FDA Trial registry, reference lists and systematic reviews for eligible studies. Additional file shows the detailed search strings (see Additional file 1: Search strings).

### Eligibility

We included randomized clinical trials (RCTs), quasi-randomized studies, and observational studies with no restrictions to language, publication date or journal, and meeting the following criteria: Patients of ≥ 18 years of age, admission to an intensive care unit (ICU), and presenting data on delirium motor subtype distribution. Exclusion criteria were: no full text obtainable (abstracts only, preprints, and conference proceedings were excluded), studies only including patients with delirium tremens (alcohol withdrawal delirium), and studies conducted in a post-anesthesia care unit.

### Delirium and motor subtype definition

The diagnosis of delirium was determined by methods as described in the included studies (see Additional file 1: Table S2). For studies using more than one assessment method/tool (e.g., in studies comparing two or more tools), we prioritized the results as follows: 1. Diagnostic and Statistical Manual for Mental Disorders (DSM III-V), 2. The Confusion Assessment Method for the ICU (CAM-ICU), and 3. Intensive Care Delirium Screening Checklist (ICDSC). Definition of the delirium motor subtype was also determined by methods as described in the included studies, whether these were based on sedation/agitation scales (such as Richmond Agitation and Sedation Scale (RASS)) or the clinical presentation of the patient.

### Study selection and data extraction

Six authors (KC, NA, LMP, SW, MOC, and CBM), working in pairs, screened titles and abstracts, and extracted data from included full text studies. Disagreements were solved by consensus or by involving OM. We used EndNote version X9, Covidence (https://www.covidence.org), and Microsoft Excel to manage records, data, and de-duplicate references. Extracted data are listed in Additional file (see Additional file 1: List of extracted data). For missing or incomplete information, we contacted the corresponding authors by email.

### Outcome measures

Our primary outcome was the distribution of delirium motor subtypes. Distribution was defined as the proportion of hypoactive, hyperactive, and mixed delirium in the pooled delirious cohort. Mixed delirium was defined as any combination of hypo- and hyperactive delirium assessments during ICU stay. We did not distinguish between incidence and prevalence when assessing the distribution of delirium motor subtypes. Delirium incidence and prevalence for the pooled cohort were secondary outcomes. We defined the incidence of delirium as being newly developed delirium in the ICU and prevalence of delirium as being all diagnosed delirium cases in the ICU. We performed a sensitivity analysis comparing incidence and prevalence of delirium between studies including versus excluding comatose patients. For each delirium motor subtype, we reported the following secondary outcomes: delirium duration, pharmacological interventions, hospital and ICU length of stay, and ICU and hospital mortality. Studies mainly reported unspecific delirium treatment strategies, such as following the PADIS guideline [20]. We were unable to retrieve data on specific agents used for delirium treatment; however, some studies reported a pharmacological treatment strategy for delirium and specific agents given to delirious patients during ICU, but with no indication reported. Consequently, we reported pharmacological interventions in terms of *delirium targeted pharmacological strategy,* defined as number of patients intervened with a pharmacological delirium treatment strategy, and *administration of specific agents* defined as reported administration of antipsychotics, α2-agonists, benzodiazepines, and propofol with no limits to indication. We chose these agents as antipsychotics, α2-agonists, and benzodiazepines, which are the most commonly used agents, for delirium treatment [21]. Propofol was added since this agent is frequently used to sedate delirious patients. We excluded RCTs in the analyses of pharmacological interventions.

### Critical appraisal

We used Cochrane Collaboration Risk of Bias Tool [22] to evaluate the risk of systematic errors of included RCTs as either high, unclear, or low risk of bias. For evaluation of the quality of included observational studies, we used the National Institutes of Health (NIH) Quality Assessment Tools [23] with adjudication of either poor, unclear, or good quality.

### Statistical analysis

For analytic reasons, we converted values reported in medians and range or interquartile range to mean and standard deviation values according to Luo et al. and Shi et al. [24, 25]. The pooled estimates of primary and secondary outcomes were computed with meta-regression analysis using a random effect model and reported with corresponding 95% confidence intervals. The reported proportions were untransformed. Between-study variance was determined with the restricted maximum-likelihood estimator. We created forest plots and used I^2^ statistic to evaluate between-study heterogeneity and Cochrane’s Q test to establish significance of heterogeneity [26]. To evaluate differences in delirium incidence and prevalence in studies excluding and including comatose patients, we performed a sensitivity analysis. Coma was defined as stated in studies. To address confounding variables and effect modifiers, we performed a pre-planned subgroup analysis with meta-regression and stratification to estimate whether the distribution of delirium motor subtypes was dependent on age, type of ICU admission, mechanical ventilation, disease severity, inclusion/exclusion of comatose patients, or RoB/quality assessment (QA). The disease severity was divided into high or low severity (high disease severity was a priori defined as a predicted risk of mortality of ≥ 50%: APACHE II ≥ 21, SAPS II ≥ 55, SAPS III ≥ 70, SOFA score ≥ 11). If a study reported multiple severity of illness scores, the study was rated corresponding to the score predicting the highest mortality. For subgroup analysis, we assumed a common between-study variance component. All statistical analyses were performed in R Statistical Software (https://www.r-project.org/) version 4.0.1. [27] using the meta- and metafor package.

## Results

### Search and study characteristics

**Fig. 1:**
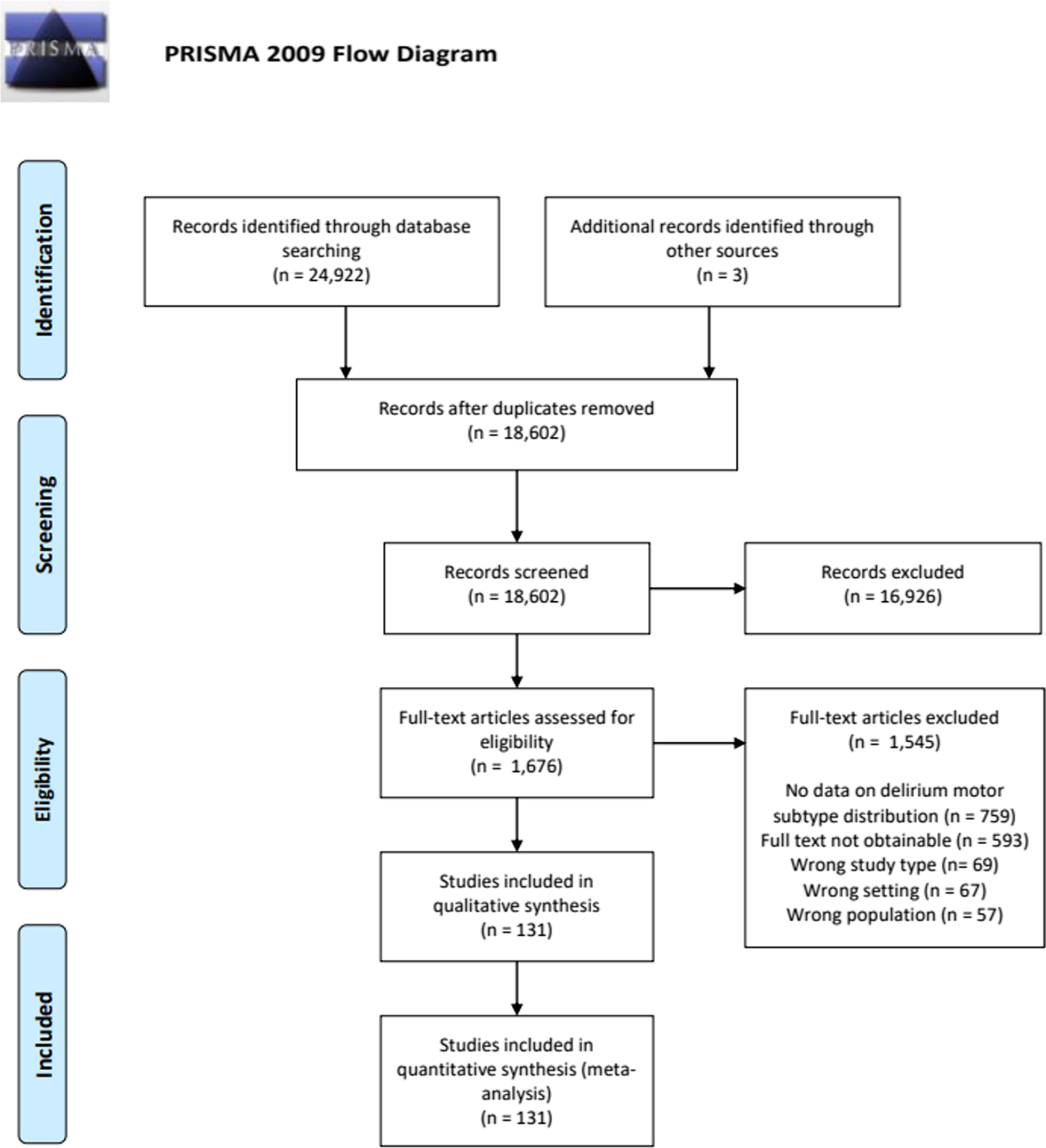
PRISMA flow diagram

Our initial literature search identified 18,602 studies. After screening and full text review, a total of 131 studies were included comprising a pooled cohort of 50,232 patients. We mainly excluded studies due to the lack of data on delirium motor subtype distribution or no full text obtainable (Fig. 1). Included studies differentiated notably in study design, setting, population, and outcome reporting. Randomized controlled studies (RCTs) and case–control studies typically did not report on delirium incidence nor prevalence. Characteristics of included studies are presented in Table 1. We contacted 125 authors via email, 37 answered, and 19 provided additional data. Characteristics of the total pooled cohort (see Additional file 1: Table S3) as well as an overview of included studies (see Additional file 1: Table S1) can be found in Additional file.

**Table 1:** Characteristics of included studies

Study characteristics	No. of studies, *n* (%)
Total number of included studies	131 (100)
Study design	
Prospective cohort study	96 (73.3)
Retrospective cohort study	14 (10.7)
Case–control study	6 (4.6)
Randomized controlled trial	9 (6.9)
Other*	6 (4.6)
Publication year	
2001–2005	2 (1.5)
2006–2010	15 (11.5)
2011–2015	42 (32.1)
2016–2020	72 (55.0)
Type of ICU	
Surgical	23 (17.6)
Medical	12 (9.2)
Mixed	53 (40.5)
Cardiac	35 (26.7)
Other	8 (6.1)
Cochrane Risk of bias/NIH quality**	
High/poor	58 (44.3)
Unclear/unclear	39 (29.8)
Low/good	34 (26.0)

*RCT* randomized clinical trial, *ICU* intensive care unit

^*^Before/after studies and quasi-randomized studies

^**^Cochrane RoB was used for RCTs and NIH quality assessment tools were used for observational studies

### Delirium incidence and prevalence

The pooled incidence of delirium was 22.2% [95% CI 18.6–25.8], and the pooled prevalence was 38.3% [95% CI 34.2–42.4]. Forrest plots in Additional file show delirium incidence and prevalence across included studies (see Additional file 1: Figs. S1 and S2). To diagnose delirium, CAM-ICU was the most frequently used method (87.0%) followed by DSM-III–V (18.3%) and ICDSC (12.9%) and most studies defined motor subtype with RASS (86.3%). Some studies used more than one tool. On average, patients were assessed for delirium 1.7 times a day, and in most studies delirium assessments were performed by nurses throughout ICU stay (see Additional file 1: Table S2)*.* In the sensitivity analyses comparing studies including versus excluding comatose patients, we found no significant difference in prevalence and incidence of delirium.

### The delirious patient cohort

The pooled delirious cohort comprised of 13,902 patients. The mean age of patients with delirium was 66.9 years [95% CI 65.4–68.5]. Disease severity scores were primarily reported by APACHE II score (44.3%), and the mean score for the delirious cohort was 18.9 [95% CI 17.5–20.3]. Patients with delirium were intervened with a delirium-targeted pharmacological strategy in 66.1% [95% CI 55.4–76.8] of cases. The investigated pharmacological agent that was most frequently administrated to delirious patients was antipsychotics (49.6% [95% CI 39.2–60.0]) followed by propofol (42.4% [95% CI 28.7–56.2]), benzodiazepines (39.8% [95% CI 31.2–48.5]), and α2-agonists (26.3% [95% CI 17.4–35.1]). The mean length of stay for patients with delirium was 9.2 days [95% CI 8.0–10.4] in the ICU and 19.8 days [95% CI 17.6–22.1] in the hospital. The ICU mortality for the delirious cohort was 17.0% [95% CI 12.6–21.4], and the hospital mortality was 21.3% [95% CI 14.0–28.6]. Table 2 presents the characteristics of the pooled delirious cohort.

**Table 2:** Characteristics of patients with delirium

Characteristics of patients with delirium (*n* = 13,902)	Studies reporting on outcome (*n*)	Delirious patients in studies reporting on outcome (*n*)	Pooled mean or pooled proportion	95% CI
Age	96	12,112	66.9 years	65.4–68.5
Sex (male)	91	11,637	62.1%	60.2–64.0
Severity of disease				
APACHEII	48	5897	18.9	17.5–20.3
SAPSII	10	1206	42.7	37.0–48.4
SAPSIII	2	285	52.2	33.2–71.2
SOFA	16	2046	7.8	6.3–9.3
Delirium-targeted pharmacological strategy*	21	3979	66.1%	55.4–76.8
Administration of specific agents**				
Antipsychotics	33	5233	49.6%	39.2–60.0
α2-agonists	24	2474	26.3%	17.4–35.1
Benzodiazepines	38	3843	39.8%	31.2–48.5
Propofol	18	2057	42.4%	28.7–56.2
Length of stay				
ICU	67	9813	9.2 days	8.0–10.4
Hospital	42	7889	19.8 days	17.6–22.1
Mortality				
ICU	29	4519	17.0%	12.6–21.4
Hospital	18	1909	21.3%	14.0–28.6

*APACHE* Acute Physiology and Chronic Health Evaluation, *SAPS* Simplified Acute Physiology Score, *SOFA* Sequential Organ Failure Assessment

^*^Patients intervened with a delirium-targeted pharmacological treatment strategy during ICU stay

^**^Patients receiving administration of specific agents (as listed) while in the ICU with no limits to indication

### Distribution of delirium motor subtypes

One hundred and twelve studies contributed to the distribution of all three motor subtypes, while 19 studies contributed to the distribution of hypoactive and hyperactive delirium only. Dichotomous distribution of delirium motor subtypes was typically reported in studies determining delirium motor subtype upon study inclusion making it impossible to determine the mixed subtype. Among studies reporting on all three motor subtypes, we found that hypoactive delirium was the most prevalent (50.3% [95% CI 46.0–54.7]), followed by mixed delirium (27.7% [95% CI 24.1–31.3]) and hyperactive delirium (22.7% [95% CI 19.0–26.5]). Data on distribution of delirium motor subtypes are presented in Table 3. Forrest plots in Additional file illustrate the prevalence of the three motor subtypes across studies (see Additional file 1: Figs. S3, S4, and S5).

**Table 3:** Distribution of delirium motor subtypes in studies with 3 or 2 motoric subtypes

Delirium motor subtype	Studies reporting on outcome (*n*)	Delirious patients in studies reporting on outcome (*n*)	Pooled proportion (%)	95% CI
Distribution of delirium motor subtypes				
Studies reporting 3 motoric subtypes				
Hypoactive*	111	11,663	50.3	46.0–54.7
Hyperactive**	109	11,626	22.7	19.0–26.5
Mixed	108	11,509	27.7	24.1–31.3
Studies reporting 2 motoric subtypes***				
Hypoactive	19	2434	61.4	49.2–73.5
Hyperactive	19	2434	38.6	26.5–50.8

^*^Three studies only had data on distribution of the hypoactive delirium motor subtype

^**^One study only had data on distribution of the hyperactive delirium motor subtype

^***^These studies only discriminated between hypoactive and hyperactive delirium motor subtype

### Secondary outcomes

Few studies reported on secondary outcomes at motor subtype level and hence contributed to these results. Patients with mixed delirium motor subtype had the longest delirium duration (days) (mixed: 3.6 [95% CI 2.6–4.5], hypo: 2.4 [95% CI 1.9–2.8], hyper: 2.2 [95% CI 1.7–2.7]), ICU length of stay (days) (mixed: 10.3 [95% CI 7.7–12.9], hypo: 8.4 [95% CI 6.4–10.5], hyper: 6.9 [95% CI 5.1–8.8]), and hospital length of stay (days) (mixed: 25.1 [95% CI 18.3–31.9], hypo: 20.0 [95% CI 15.8–24.2], hyper: 18.5 [95% CI 14.2–22.8]), and the highest ICU mortality (%) (mixed: 30.0 [95% CI 14.1–45.9], hypo: 27.9 [95% CI 17.5–38.5], hyper: 21.8 [95% CI 9.4–34.2]), and hospital mortality (%) (mixed: 32.8 [95% CI 15.3–50.4], hyper: 30.1 [95% CI 4.8–57.1], hypo: 27.2 [95% CI 10.1–44.3]). Patients with mixed delirium were more frequently received antipsychotics (%) (58.5 [95% CI 34.2–82.9]), α2-agonists (53.7 [95% CI 25.4–82.0]), benzodiazepines (54.1 [95% CI 29.6–78.7]), and propofol (67.7 [39.3–96.1]) than other delirium motor subtypes. Patients with hyperactive delirium were more frequently intervened with a delirium-targeted pharmacological strategy compared to patients with mixed or hypoactive delirium (%) (63.7 [95% CI 39.8–87.5] vs. 58.3 [95% CI 34.2–82.9] vs. 38.8 [95% CI 17.0–60.7]). For data on secondary outcomes (delirium duration, length of stay, mortality, and pharmacological interventions), see Table 4.

**Table 4:** Secondary outcomes

Delirium motor subtype	Studies reporting on outcome (*n*)	No. of patients with outcome (*n*)	Pooled mean/proportion	95% CI
Delirium duration				
Hypoactive	26	1589	2.4 days	1.9–2.8
Hyperactive	25	709	2.2 days	1.7–2.7
Mixed	23	1591	3.6 days	2.6–4.5
ICU length of stay				
Hypoactive	25	1707	8.4 days	6.4–10.5
Hyperactive	22	541	6.9 days	5.1–8.8
Mixed	22	1513	10.3 days	7.7–12.9
Hospital length of stay				
Hypoactive	19	1596	20.0 days	15.8–24.2
Hyperactive	16	545	18.5 days	14.2–22.8
Mixed	17	1670	25.1 days	18.3–31.9
ICU mortality				
Hypoactive	12	779	27.9%	17.5–38.3
Hyperactive	9	313	21.8%	9.4–34.2
Mixed	9	685	30.0%	14.1–45.9
Hospital mortality				
Hypoactive	4	305	27.2%	10.1–44.3
Hyperactive	3	106	30.1%	4.8–57.1
Mixed	4	305	32.8%	15.3–50.4
Delirium-targeted pharmacological strategy*				
Hypoactive	7	358	38.8%	17.0–60.7
Hyperactive	7	258	63.7%	39.8–87.5
Mixed	7	479	58.3%	32.4–84.3
Administration of antipsychotics**				
Hypoactive	9	885	39.4%	16.0–62.9
Hyperactive	8	321	56.2%	31.6–80.8
Mixed	9	1073	58.5%	34.2–82.9
Administration of α2-agonists**				
Hypoactive	7	214	22.1%	10.1–34.1
Hyperactive	7	188	33.4%	13.5–53.3
Mixed	7	275	53.7%	25.4–82.0
Administration of benzodiazepines**				
Hypoactive	7	237	31.9%	16.5–47.2
Hyperactive	7	194	28.0%	21.8–34.1
Mixed	7	251	54.1%	29.6–78.7
Administration of propofol**				
Hypoactive	6	188	53.1%	31.0–75.2
Hyperactive	6	164	56.0%	28.1–83.8
Mixed	6	242	67.7%	39.3–96.1

^*^Patients intervened with a delirium-targeted pharmacological treatment strategy during ICU stay

^**^Patients receiving administration of this specific agents while in the ICU with no limits to indication

### Subgroup analysis

In the pre-planned subgroup analysis, we found that hypoactive delirium was significantly more prevalent in studies with older delirious patients (mean age ≥ 65 years) compared to studies with younger delirious patients (mean age < 65 years) (%) (54.0 [95% CI 48.5–59.5] vs. 40.0 [95% CI 32.7–47.2]) (*p* = 0.002). Mixed delirium was significantly more prevalent in studies with younger delirious patients (mean age < 65 years) compared to studies with older delirious patients (mean age ≥ 65 years) (%) (35.8 [95% CI 29.0–42.6] vs. 23.8 [95% CI 18.7–28.9]) (*p* = 0.006). The prevalence of mixed delirium was significantly higher amongst studies with low risk of bias or good quality compared to studies with high risk of bias or poor quality (%) (33.4 [95% CI 26.1–40.7] vs. 23.4 [95% CI 17.8–29.0]) (*p* = 0.03). Distribution of delirium motor subtypes was not dependent on the type of ICU (medical vs. surgical and cardiac vs. other ICUs), mechanical ventilation, including vs. excluding comatose patients or severity of disease. Table 5 presents the subgroup analysis.

**Table 5:** Subgroup analysis

Subgroup (no. of studies in this subgroup)	Hypoactive pooled proportion (%) (95% CI)	Hyperactive pooled proportion (%) (95% CI)	Mixed pooled proportion (%) (95% CI)
Subgroup analysis			
Medical versus surgical ICUs			
Medical (*n* = 12)	40.4 (24.8–55.9)	28.2 (15.6–40.7)	29.4 (17.6–41.1)
Surgical (*n* = 23)	54.1 (43.9–64.3)	23.7 (15.3–32.1)	26.3 (18.3–34.2)
Cardiac versus other ICUs			
Cardiac (*n* = 35)	51.2 (43.1–60.3)	28.5 (21.2–35.8)	21.7 (14.7–28.8)
Other (*n* = 96)	50.0 (44.9–55.0)	20.7 (16.4–25.0)	29.5 (25.4–33.6)
Mean age			
< 65 (*n* = 38)	40.0 (32.7–47.2)*	23.2 (16.5–29.9)	35.8 (29.0–42.6)*
≥ 65 (*n* = 60)	54.0 (48.5–59.5)* *p* = 0.002	22.6 (17.6–27.7)	23.8 (18.7–28.9)* *p* = 0.006
Mechanical ventilation			
≤ 20% MV (*n* = 13)	53.7 (38.3–69.1)	27.1 (15.8–38.4)	24.6 (10.0–39.2)
≥ 80% MV (*n* = 38)	48.9 (40.4–57.3)	17.3 (11.5–23.0)	32.7 (25.2–40.3)
Disease severity			
Low (*n* = 58)	51.3 (45.3–57.3)	21.1 (16.7–25.4)	29.2 (23.8–34.6)
High (*n* = 21)	49.8 (39.3–60.3)	16.9 (9.5–24.4)	32.1 (22.7–41.4)
Comatose patients			
Including (*n* = 40)	63.7 (46.7–60.8)	21.5 (15.4–27.6)	27.4 (21.3–33.5)
Excluding (*n* = 65)	48.4 (42.8–54.0)	22.1 (17.4–26.9)	28.3 (23.6–32.9)
Risk of bias/quality assessment			
Low RoB/good QA (*n* = 34)	53.3 (44.5–62.1)	18.1 (10.4–25.8)	33.4 (26.1–40.7)*
High RoB/Poor QA (*n* = 58)	50.2 (43.1–57.2)	25.1 (19.1–31.2)	23.4 (17.8–29.0)* *p* = 0.03

*ICU* intensive care unit, *RoB* risk of bias, *QA* quality assessment

^*^Statistically significant difference

## Discussion

In this scoping systematic review comprising 131 studies and a pooled cohort of 50,232 ICU patients of whom 13,902 had delirium, we found hypoactive delirium to be the most prevalent delirium motor subtype among ICU patients accounting for half (50.3%) of delirium cases. Furthermore, we identified patients with mixed delirium to have the longest delirium duration, ICU and hospital length of stay, and the highest ICU and hospital mortality. Data on pharmacological interventions were complex. While patients with hyperactive delirium were more frequently intervened with a delirium-targeted pharmacological strategy, patients with mixed delirium were more likely to receive administration of specific pharmacological agents (antipsychotics, α2-agonists, benzodiazepines, and propofol) during their ICU stay (no reported indication). Our subgroup analysis identified hypoactive delirium to be more prevalent in studies with high mean age (≥ 65 years) in delirious patients and mixed delirium to be more prevalent in studies with low mean age (< 65 years) in delirious patients. The subgroup analyses also revealed a significantly higher proportion of mixed delirium cases in studies with low risk of bias or good quality.

Our reported distribution of delirium motor subtypes is in accordance with findings in a recent review by Krewulak et al. [28] who included 48 studies and 27,342 ICU patients. Krewulak et al. [28] found hypoactive delirium to have the highest mortality in four of seven included studies. In contrast, we found the highest ICU and hospital mortality in patients with mixed delirium in eight of twelve studies. These differences could be due to the excessive heterogeneity between the included studies. However, in one of the most recent and largest studies (*n* = 6323) reporting on delirium motor subtypes, the highest mortality rates and ICU length of stay were also found for patients with mixed delirium [29]. Pisani et al. [30] found an association between the number of days of delirium in the ICU and higher mortality. These findings support why mixed delirium with the longest delirium duration also has the highest mortality. In a recent study from the BRAIN-ICU cohort, hypoactive delirium was not associated with increased risk of death in the hospital [31]. Patients with mixed delirium received more pharmacological interventions than other delirium motor subtypes. An important question to ask is whether this could be associated with the increased mortality found in these patients.

Management of delirium is challenging, and no evidence-based treatment currently exists. Few studies in this review reported on pharmacological interventions between delirium motor subtypes. We found ICU patients diagnosed with hyperactive delirium to be more frequently intervened with a delirium-targeted pharmacological strategy. This is in accordance with other studies [32, 33]. However, when investigating administration of specific agents, including antipsychotics and sedatives, given to delirious patients during ICU stay, mixed delirium received more agents compared to the other motor subtypes. These findings are complex and generate multiple considerations. Antipsychotics are supported as potentially beneficial in the treatment of hyperactive delirium [20]. It could be speculated that hyperactive delirium is more reversible by responding well to treatment resulting in shorter delirium duration, ICU stay, and reduced exposure to pharmacological interventions in general. All the investigated agents could induce a hypoactive state in an initially hyperactive patient, resulting in a medically induced mixed motor subtype. The longer ICU stay reported in patients with mixed delirium, compared to the other motor subtypes, may account for some of the increased antipsychotic and sedative exposure; however, the inverse relationship is also plausible as inappropriate treatment may complicate ICU stay causing prolonged delirium duration and death. Deep sedation, agitation, and cumulative dose of benzodiazepines have been associated with increased mortality [34].

Studies in our review rated low risk of bias or good quality had a higher proportion of patients with mixed delirium. This could be due to the methodology of delirium assessment strategy used in these studies, as studies screening for delirium several times daily and for a longer period were rated higher quality and patients in these studies would have greater chance of getting both hypo- and hyperactive delirium assessments. Studies in our review screened patients for delirium averagely 1.7 times a day, which is less than the recommended 2–3 times daily (once every shift or 8/12 h) [20]. Delirium is a transient condition so when delirium screening does not occur frequently one might miss hyper- or hypoactive periods in a delirious patient, hence misdiagnosing the delirium motor subtype. If mixed delirium is more common in studies that have increased assessments, then it is likely more common than most of the literature would suggest. However, with current knowledge it is impossible to determine whether mixed delirium is its own pathologic entity or simply present in patients experiencing disease entities of both hypoactive and hyperactive delirium combined.

## Strengths and limitations

We included both RCTs and observational studies, which enabled us to investigate and describe the distribution of delirium motor subtypes in ICU patients independent of study design. Although making comparisons more difficult, this strengthens data quality and furthermore resulted in including the largest number of studies and largest pooled cohort evaluating distribution and outcomes of delirium motor subtypes to date.

This review also has limitations. First, heterogeneity in methodology and in outcome reporting amongst included studies was frequent. Second, not all studies differentiated between incidence and prevalence of delirium. Some studies used the term ‘incidence of delirium’ but did not account for having excluded patients with onset of delirium before ICU admission. Third, different methods for screening and diagnosis of delirium were applied across studies, and even though our review concludes that most studies applied CAM-ICU to diagnose delirium, the tool was not always used identically. Fourth, 74.1% of the included studies suffered from poor or unclear quality making baseline risk for confounding high at the point of data collection. Fifth, research on delirium motor subtypes mainly report low prevalence of pure hyperactive delirium. This is reflected in the wide confidence intervals surrounding ICU and hospital mortality in the hyperactive delirium cohort. Sixth, some studies defined mixed delirium as having both hypo- and hyperactive delirium assessments during the same day. Unless the authors could provide data on distribution of delirium motor subtype as defined in present review, the study was excluded. Seventh, propofol is often used to sedate agitated delirious patients and hence patients with hyperactive delirium. Propofol was unlikely used as delirium treatment but rather as part of a sedation strategy in hypoactive cases.

### Perspective

It is important to clarify whether critically ill patients benefit from delirium motor subtype targeted treatments. Existing literature on outcomes related to delirium motor subtypes is poor and of low quality. Further studies are needed to determine whether short- and long-term outcomes are dependent on delirium motor subtypes and whether the subtypes react differently to treatments. Additionally, research on delirium motor subtypes in the ICU needs more strict standardization of exactly how, how often, and for how long a period delirium screening should occur. Since it is uncertain whether mixed delirium is truly its own disease entity, or just a mix hypoactive and hyperactive delirium episodes, future studies should consider time spent in each subtype for each patient, to examine whether one or the other is associated with worse outcomes.

## Conclusions

In this review on distribution of delirium motor subtypes in the ICU, we found that hypoactive delirium was the most prevalent delirium motor subtype accounting for approximately half of delirium cases. Patients with mixed delirium had longer delirium duration, ICU and hospital length of stay, and higher ICU and hospital mortality than the other delirium motor subtypes. Patients with mixed delirium were more likely to receive administration of antipsychotics, α2-agonists, benzodiazepines, and propofol while in the ICU (no indication reported), while patients with hyperactive delirium were more likely to be intervened with a delirium-targeted pharmacological strategy. The identified differences among delirium motor subtypes in the ICU should be further investigated as they could be the key to future improvement of delirium care. The large between-study heterogeneity suggests that a more standardized methodology in delirium research is warranted.

## Supplementary Information

**Additional file 1.** Search strings; **Table S1.** Overview of included studies; List of extracted data; **Forrest plots: Fig. S1.** Delirium incidence, **Fig. S2.** Delirium prevalence, **Fig. S3.** Distribution of hypoactive delirium, **Fig. S4.** Distribution of hyperactive delirium, **Fig. S5.** Distribution of mixed delirium; **Table S2.** Delirium assessment methods; **Table S3.** Characteristics of total cohort. **Description of data.** The data in the Additional file provides the reader with detailed search strings, a table with an overview of included studies with main characteristics and a reference for each study, a list of data we intended to extract for each study, forest plots of incidence and prevalence delirium and distribution of hypoactive, hyperactive and mixed delirium, a table showing data regarding delirium assessment methods used in included studies, and a table showing characteristics of the total pooled cohort in studies included.

## Data Availability

The datasets used and/or analyzed during the current study are available from the corresponding author on reasonable request.

## Abbreviations

APACHE: Acute Physiology and Chronic Health Evaluation
CAM-ICU: The Confusion Assessment Method for the ICU
DSM: Diagnostic and Statistical Manual for Mental Disorders
ICDSC: Intensive Care Delirium Screening Checklist
ICU: Intensive care unit
NIH: National Institutes of Health
PRISMA-ScR: Preferred Reporting Items for Systematic reviews and Meta-Analyses extension for Scoping Reviews
QA: Quality assessment
RASS: Richmond Agitation and Sedation Scale
RCT: Randomized controlled trial
SAPS: Simplified Acute Physiology Score
SOFA: Sequential Organ Failure Assessment
