# Supplementary material for: Distribution of delirium motor subtypes in the intensive care unit: a systematic scoping review
Source: Crit Care. 2022 Mar 3;26:53. doi: 10.1186/s13054-022-03931-3 (PMC8896322; doi:10.1186/s13054-022-03931-3)
Supplement: Supplementary file 1 — Additional file 1. Search strings; Table S1. Overview of included studies; List of extracted data; Forrest plots: Fig. S1. Delirium incidence, Fig. S2. Delirium prevalence, Fig. S3. Distribution of hypoactive delirium, Fig. S4. Distribution of hyperactive delirium, Fig. S5. Distribution of mixed delirium; Table S2. Delirium assessment methods; Table S3. Characteristics of total cohort. Description of data. The data in the Additional file provides the reader with detailed search strings, a table with an overview of included studies with main characteristics and a reference for each study, a list of data we intended to extract for each study, forest plots of incidence and prevalence delirium and distribution of hypoactive, hyperactive and mixed delirium, a table showing data regarding delirium assessment methods used in included studies, and a table showing characteristics of the total pooled cohort in studies included. [file 13054_2022_3931_MOESM1_ESM.docx]

**Distribution of delirium motor subtypes in the intensive care unit:**

**A systematic scoping review**

Kirstine N. la Cour^1^, Nina C. Andersen-Ranberg^1^, Sarah Weihe^1^, Lone M. Poulsen^1^, Camilla B. Mortensen^1^, Cilia K. W. Kjer^1^, Marie O. Collet^2^, Stine Estrup^1^, Ole Mathiesen^1,3^.

^1^ Centre for Anaesthesiological Research, Department of Anesthesiology, Zealand University Hospital, Koege, Denmark

^2^ Department of Intensive Care, Copenhagen University Hospital, Rigshospitalet, Denmark.

^3^ Department of Clinical Medicine, Copenhagen University, Denmark.

Contents

[Search Strings 2](#_Toc88925116)

[Overview of included studies 7](#_Toc88925117)

[List of extracted data 25](#_Toc88925118)

[Forest plots 26](#_Toc88925119)

[Delirium Assessment Methods 31](#_Toc88925120)

[Characteristics of total cohort 32](#_Toc88925121)

# Search Strings

Each search in each database consists of a search based on subject headings and a search based on free text. When the search was conducted all hits/references were imported to *Covidence* and duplicates were removed. The following describes for each database:

- Title of database
- Search via subject headings / free text
- Date for when the search was performed
- Number of duplicates removed when imported to Covidence
- Number of references imported to Covidence to be screened
- Exact phrase as put in the search field
- Renewed search details

**PubMed systematic search using MeSH (7714 hits)**

Performed April 15^th^ 2020

Duplicates removed: 14

Number of references to be screened: 7700

"Delirium"[Mesh] OR "Sepsis-Associated Encephalopathy"[Mesh] OR "Neurocognitive Disorders"[Mesh] OR "Consciousness Disorders"[Mesh] OR "Stupor"[Mesh] OR "Psychotic Disorders"[Mesh] OR "Cognition Disorders"[Mesh] OR "Cognitive Dysfunction"[Mesh] OR "Psychomotor Disorders"[Mesh] OR "Psychomotor Agitation"[Mesh]

OR

Delirium[Title/Abstract] OR Sepsis-Associated Encephalopathy[Title/Abstract] OR Neurocognitive Disorders[Title/Abstract] OR Consciousness Disorders[Title/Abstract] OR Stupor[Title/Abstract] OR Psychotic Disorders[Title/Abstract] OR Cognition Disorders[Title/Abstract] OR Cognitive Dysfunction[Title/Abstract] OR Psychomotor Disorders[Title/Abstract] OR Psychomotor Agitation[Title/Abstract] OR Delirium Restlessness[Title/Abstract] OR Psychomotor Hyperactivity[Title/Abstract] OR Psychomotor Restlessness[Title/Abstract] OR Psychomotor Excitement[Title/Abstract] OR Psychomotor Impairment[Title/Abstract] OR Cognitive Impairment[Title/Abstract] OR Cognitive Decline[Title/Abstract] OR Mental Deterioration[Title/Abstract] OR Psychosis[Title/Abstract] OR Psychoses[Title/Abstract] OR Nonpsychotic Organic Brain Syndrome[Title/Abstract] OR Organic Mental Disorder[Title/Abstract] OR Sepsis Associated Encephalopathy[Title/Abstract] OR Sepsis-Associated Delirium[Title/Abstract] OR Sepsis Associated Delirium[Title/Abstract] OR Subacute Delirium[Title/Abstract] OR Mixed Origin Delirium[Title/Abstract]

AND

("Intensive Care Units"[Mesh] OR "Critical Care"[Mesh] OR "Critical Illness"[Mesh] OR "Respiration, Artificial"[Mesh] OR “Critical Care Nursing"[Mesh]) NOT ("Intensive Care Units, Pediatric"[Mesh] OR "Intensive Care Units, Neonatal"[Mesh])

OR

(Intensive Care Unit[Title/Abstract] OR Critical Care[Title/Abstract] OR Critical Illness[Title/Abstract] OR Respiration, Artificial[Title/Abstract] OR Intensive Care[Title/Abstract] OR Critically Ill[Title/Abstract] OR Mechanical Ventilation[Title/Abstract]) NOT (Pediatric OR Neonatal)

**PubMed free text search (752 hits)**

Performed April 15^th^ 2020

Duplicates removed: 534

Number of references to be screened: 218

Delirium OR delirious OR confusion OR disorientation OR "acute confusional state" OR "acute encephalopathy" OR "acute cognitive dysfunction" OR ”acute cognitive impairment” OR "ICU psychosis" OR "acute brain dysfunction" OR "acute brain failure" OR "intensive care unit syndrome" OR “ICU syndrome”

AND

”Intensive care unit” OR ICU OR ”critical care” OR ”intensive care” OR ”critically ill” OR “critical illness” OR "mechanical ventilation" OR "mechanically ventilated”

AND

Type* OR subtype* OR hyperactive* OR hypoactive* OR mixed OR agitated OR lethargic OR motor*

**Ovid/EMBASE systematic search using Emtree (9465 hits)**

Performed March 20^th^ 2020

Duplicates removed: 2774

Number of references to be screened: 6651

delirium/ or acute confusion/ or sepsis associated encephalopathy/ or stupor/ or acute psychosis/ or intensive care psychosis/ or restlessness/ or hyperactivity/ or hypoactivity/ or attention disturbance/ or cognitive defect/ or confusion/ or disorientation/ or organic brain syndrome/ or organic psychosyndrome/ or lethargy/ or thought disorder/ or thinking impairment/ or abnormal thinking/

AND

(intensive care/ or artificial ventilation/ or intensive care nursing/ or intensive care unit/ or medical intensive care unit/ or neurological intensive care unit/ or surgical intensive care unit/or critical illness/) not (pediatric.mp or neonatal.mp)

**Ovid/EMBASE free text search (1698 hits)**

Performed April 16^th^ 2020

Duplicates removed: 560

Number of references to be screened: 1138

(Delirium or delirious or confusion or disorientation or acute confusional state or acute encephalopathy or acute cognitive dysfunction or acute cognitive impairment or ICU psychosis or acute brain dysfunction or acute brain failure or intensive care unit syndrome or icu syndrome).mp

and

(Intensive care unit or ICU or critical care or intensive care or critically ill or mechanical ventilation or mechanically ventilated).mp

and

(Type* or subtype* or hyperactive* or hypoactive* or mixed or agitated or lethargic or motor*).mp

**Cinahl systematic search (1932 hits)**

Performed April 17^th^ 2020

Duplicates removed: 1213

Number of references to be screened: 719

(MH "Delirium") OR (MH "ICU Psychosis") OR (MH "Organic Mental Disorders, Psychotic+") OR (MH "Delusions+") OR (MH "Organic Mental Disorders+") OR (MH "Confusion+") OR (MH "Consciousness Disorders") OR (MH "Cognition Disorders+") OR (MH "Confusion+") OR (MH "Acute Confusion (NANDA)") OR (MH "Agitation") OR (MH "Delusions") OR (MH "Psychomotor Disorders+") OR (MH "Psychomotor Agitation+") OR (MH "Neurobehavioral Manifestations+")

AND

(MH "Intensive Care Units+") NOT (MH "Intensive Care Units, Neonatal" OR MH "Intensive Care Units, Pediatric+")

**Cinahl free text search (365 hits)**

Performed April 17^th^ 2020

Duplicates removed: 318

Number of references to be screened: 47

Delirium OR delirious OR confusion OR disorientation OR "acute confusional state" OR "acute encephalopathy" OR "acute cognitive dysfunction" OR ”acute cognitive impairment” OR "ICU psychosis" OR "acute brain dysfunction" OR "acute brain failure" OR "intensive care unit syndrome" OR “ICU syndrome”

AND
(”Intensive care unit” OR ICU OR ”critical care” OR ”intensive care” OR ”critically ill” OR “critical illness” OR "mechanical ventilation" OR "mechanically ventilated”)

AND
Type* OR subtype* OR hyperactive* OR hypoactive* OR mixed OR agitated OR lethargic OR motor*

**PsychINFO systematic search using thesauruses (240 hits)**

Performed April 17^th^ 2020

Duplicates removed: 186

Number of references to be screened: 54

DE "Delirium" OR DE "Consciousness Disturbances" OR DE "Neurocognitive Disorders" OR DE "Place Disorientation" OR DE "Time Disorientation" OR DE "Consciousness Disorders" OR DE "Thought Disturbances" OR DE "Delusions" OR DE "Cognitive Impairment" OR DE "Mental Confusion" OR DE "Organic Brain Syndromes" OR DE "Toxic Encephalopathies" OR DE "Encephalopathies" OR DE "Toxic Psychoses" OR DE "Acute Psychosis" OR DE "Restlessness" OR DE "Agitation"

AND

(DE "Intensive Care")

**PsychINFO free text search (159 hits)**

Performed April 17^th^ 2020

Duplicates removed: 105

Number of references to be screened: 54

Delirium or delirious or confusion or disorientation or acute confusional state or acute encephalopathy or acute cognitive dysfunction or acute cognitive impairment or ICU psychosis or acute brain dysfunction or acute brain failure or intensive care unit syndrome or ICU syndrome

AND

Intensive care unit or ICU or critical care or intensive care or critically ill or mechanical ventilation or mechanically ventilated

AND

Type* or subtype* or hyperactive* or hypoactive* or mixed or agitated or lethargic or motor*

**Cochrane systematic search using MeSH (125 hits)**

Performed April 17^th^ 2020

Duplicates removed: 125

Number of references to be screened: 0

[Delirium] OR [Confusion] OR [Consciousness Disorders] OR [Lethargy] OR [Neurobehavioral Manifestations] OR [Psychomotor Disorders] OR [Psychomotor Agitation] OR [Sepsis-Associated Encephalopathy] OR [Psychotic Disorders] OR [Stupor] OR [Cognition Disorders] OR [Cognitive Dysfunction]

AND

([Critical Care] OR [Respiration, Artificial] OR [Intensive Care Units] OR [Ventilators, Mechanical]) NOT ([Intensive Care, Neonatal] OR [Intensive Care Units, Pediatric])

**Cochrane free text search (375 hits)**

Performed April 17^th^ 2020

Duplicates removed: 162

Number of references to be screened: 213

Delirium OR delirious OR confusion OR disorientation OR "acute confusional state" OR "acute encephalopathy" OR "acute cognitive dysfunction" OR ”acute cognitive impairment” OR "ICU psychosis" OR "acute brain dysfunction" OR "acute brain failure" OR "intensive care unit syndrome" OR “ICU syndrome”

AND

”Intensive care unit” OR ICU OR ”critical care” OR ”intensive care” OR ”critically ill” OR “critical illness” OR "mechanical ventilation" OR "mechanically ventilated”

AND

Type* OR subtype* OR hyperactive* OR hypoactive* OR mixed OR agitated OR lethargic OR motor*

**Google Scholar (301 hits)**

Performed April 20^th^ 2020

Duplicates removed: 101

Number of references to be screened: 200

“intensive care unit” “delirium subtype”

**Renewed search performed April 14^th^ 2021**

**PubMed systematic search**

- 731 hits
- 38 duplicates removed
- 693 imported to Covidence

**PubMed free text search:**

- 92 hits
- 3 duplicated removed
- 89 imported to Covidence

**EMBASE/Ovid systematic search**

- 171 hits
- 16 duplicates
- 155 imported to Covidence

**EMBASE/Ovid free text search**

- 568 hits
- 50 duplicates
- 518 imported to Covidence

**CINAHL systematic search**

- 137 hits
- 72 duplicates removed
- 65 imported to Covidence

**CINAHL free text search**

- 30 hits
- 20 duplicates removed
- 10 imported to Covidence

**PsycInfo systematic search:**

- 13 hits
- 7 duplicates removed
- 6 imported to Covidence

**PsycInfo free text search:**

- 5 hits
- 2 duplicates removed
- 3 imported to Covidence

**Cochrane systematic search:**

- 0 hits

**Cochrane free text search:**

- 0 hits

**Google Scholar:**

- 49 hits
- 23 duplicates removed
- 26 imported to Covidence

**Total**

Total number of duplicates: 6323

Total number of references screened in Covidence: 1859

# Overview of included studies

Table S1 Overview of included studies

| First author (year) (reference) | Country | Design | Type of ICU | Sample size  Total / Delirious | | Delirium definition (no. of assessments) | Objectives | RoB / QA* |
| --- | --- | --- | --- | --- | --- | --- | --- | --- |
| Agarwal, V. (2010) (1) | USA | Prospective cohort study | Burns | 82 | 63 | CAM-ICU (once a day) | To evaluate the prevalence of delirium in ventilated burn patients, using validated instruments, and to identify its risk factors. | Unclear |
| Aldemir, M. (2001) (2) | Turkey | Prospective cohort study | Surgical | 818 | 90 | DSM–III–R (once a day) | To investigate the predisposing factors for delirium in a surgical ICU setting. | Poor |
| Angles, E. M. (2008) (3) | USA | Prospective cohort study | Trauma | 69 | 41 | CAM-ICU + chart review (once a day) | To describe the natural history of delirium after major injury, to identify risk factors that predict the development of post injury delirium, and to compare outcome measures in subjects with and without delirium. | Good |
| Anlacan, V. M. M (2015) (4) | The Philippines | Prospective cohort study | Mixed | 107 | 31 | CAM-ICU (once or twice a day) | To determine the prevalence and risk factors associated with delirium in patients admitted in the ICUs of Philippine General Hospital, where delirium monitoring is not routinely performed. | Unclear |
| Arroyo-Novoa, C. M. (2019) (5) | USA | Prospective cohort study | Mixed | 71 | 24 | CAM-ICU (two times a day on two days per week) | To describe the occurrence, assessment, prevention, and management practices of PAD in four ICUs from the Puerto Rico Medical Center and compared findings with the 2013 PAD guidelines. | Poor |
| Artemiou, P. (2015) (6) | Slovakia | Prospective cohort study | Cardiac | 500 | 73 | CAM-ICU (twice a day) | To test the hypothesis that restoring normal sleeping patterns with a substance that is capable of resynchronizing circadian rhythm such as exogenous administration of melatonin may possibly reduce the incidence of postoperative delirium. | Poor |
| Ayazoglu, T. A. (2012) (7) | Turkey | Prospective cohort study | Cardiac | 217 | 64 | DSM-IV + CAM-ICU (NA) | To compare a variety of characteristics in patients with and without delirium and to identify risk factors associated with delirium in patients hospitalized in an ICU after cardiac surgery. | Unclear |
| Barman, A. (2018) (8) | India | Prospective cohort study | Mixed | 310 | 140 | DSM-IV + CAM-ICU + ICDSC (three assessments in a span of 4 hours on weekdays) | To evaluate the diagnostic accuracy of CAM-ICU and ICDSC among the nursing and medical staff in a multidisciplinary ICU. | Good |
| Bigatello, L. M. (2012) (9) | USA | Prospective cohort study | Surgical | 283 | 98 | CAM-ICU (once a day) | To test the hypothesis that daily administration of a diagnostic instrument for delirium in a surgical/trauma ICU decreases the time of institution of pharmacologic therapy and improves related outcomes. | Good |
| Boettger, S. (2018) (10) | Switzerland | Prospective cohort study | Surgical | 289 | 122 | DSM-IV-TR + CAM-ICU + ICDSC + DRS-R-98 (once every 8 hours) | To evaluate the concurrent validity, sensitivity and specificity, as well the positive and negative prediction of the ICDSC threshold set at a lower score of ≥3, the current standard ≥4, and an increased score of ≥5 versus a diagnosis of delirium determined with the DSM-IV-TR. | Poor |
| Breu, A. (2015) (11) | Germany | Prospective cohort study | Cardiac | 467 | 57 | ICDSC (once every 8 hours) | To evaluate the incidence of subsyndromal delirium after cardiac surgery and its impact on clinical outcomes. | Poor |
| Brown, C. H. (2016) (12) | USA | Prospective cohort study | Cardiac | 66 | 37 | CAM + CAM-ICU + DRS-R-98 + chart review (once a day) | To test the hypothesis that rigorously assessed delirium after cardiac surgical procedures would be associated with increased length of stay in the intensive care unit, hospital length of stay, and hospital charges, even after adjusting for important patient related characteristics. | Unclear |
| Bui, L. N. (2017) (13) | USA | Retrospective cohort study | Surgical | 1055 | 423 | CAM-ICU + ICD-9-CM codes (once every 12 hours) | To compare the proportions of surgical ICU patients with delirium detected using the CAM-ICU who received administrative documentation for delirium using ICD-9-CM codes, stratified by delirium motoric subtypes. | Unclear |
| Canet, E. (2019) (14) | Australia | Retrospective cohort study | Mixed | 100 | 100 | ICD-10 codes (NA) | To test the hypothesis that patient demographics, clinical phenotype, management and outcomes of patient with delirium in hospital ward patients differ from ICU patients. | Poor |
| Caruso, P. (2014) (15) | Brazil | Retrospective cohort study | Mixed | 1253 | 163 | CAM-ICU (once every 12 hours) | To compare the ICU delirium prevalence and characteristics (coma/delirium-free days, first day in delirium, and delirium motoric subtypes) of critically ill patients admitted in single- or multibed rooms. | Unclear |
| Castaño, A. M. H. (2015) (16) | Colombia | Prospective cohort study | Mixed | 102 | 22 | CAM-ICU (once every 8 hours) | To the test the hypothesis that the awake patients on mechan­ical ventilation with primary prophylaxis was associated with a lower presence of delirium during ICU stay that is on average of 7 days. | Unclear |
| Ceriana, P. (2010) (17) | Italy | Prospective cohort study | Step Down Unit | 234 | 18 | DSM-IV + ICDSC (once a day) | To assess the incidence of and risk factors for delirium and subsyndromal forms and their impact on clinical outcome in a group of patients transferred from an ICU to an SDU. | Unclear |
| Chaiwat, O. (2019) (18) | Thailand | Prospective cohort study | Surgical | 250 | 61 | CAM-ICU (once every 12 hours) | To identify the incidence of, and the risk factors for, POD in surgical ICU patients, and to determine predictive scores for the development of POD. | Good |
| Collet, M. O. (2018) (19) | Denmark | Prospective cohort study | Mixed | 1260 | 314 | CAM-ICU + ICDSC + DOS + Nu-DESC (once a day) | To assess the prevalence and variables associated with haloperidol use for delirium in ICU patients and explore any associations of haloperidol use with 90-day mortality. | Good |
| De la Varga-Martinez, O (2020) (20) | Spain | Prospective cohort study | Cardiac | 689 | 117 | CAM-ICU (once a day) | To develop and validate a preoperative clinical prediction model that evaluates the risk of delirium development in patients undergoing cardiac surgery | Good |
| Devlin, J. W. (2011) (21) | USA | RCT | Mixed | 36 | 36 | ICDSC (once every 12 hours) | To retrospectively compare duration and time to first resolution of individual delirium symptoms from the database of a randomized, double-blind, placebo-controlled study comparing quetiapine or placebo, both with haloperidol rescue, for critically ill patients with delirium. | Low |
| Eremenko, A. A. (2014) (22) | Russia | RCT | Cardiac | 60 | 60 | ICDSC (NA) | To assess efficacy and safety of dexmedetomidine for treatment of delirium in cardiac surgery. | High |
| Estrup, S. (2018) (23) | Denmark | Retrospective cohort study | Mixed | 183 | 52 | CAM-ICU + haloperidol administration (once every 12 hours) | To describe incidence of delirium, risk factors for delirium, and the association between delirium and circadian light for patients in the ICU. | Poor |
| Falsini, G. (2018) (24) | Italy | Prospective cohort study | Cardiac | 726 | 111 | CAM-ICU (once a day) | To assess prospectively the incidence, prevalence and significance of delirium in a large, homogenous cohort of elderly patients with acute cardiac diseases. | Unclear |
| Fritz, B. A. (2016) (25) | USA | Prospective cohort study | Surgical | 619 | 162 | CAM-ICU (once every 12 hours) | To determine whether the duration of intraoperative EEG suppression is associated independently with postoperative delirium. | Good |
| Garcia-Nunez, D. (2019) (26) | Switzerland | Prospective cohort study | Surgical | 289 | 122 | DSM-IV + DRS-R-98 + DMSS (once a day) | To validate a German version of the DMSS in cardiovascular–surgical intensive care patients. | Unclear |
| Gaspardo, P (2014) (27) | Italy | Prospective cohort study | Mixed | 57 | 20 | CAM-ICU (once a day) | To determine the psychometric properties of the Italian version of the CAM-ICU. | Poor |
| Girard, T. D (2018) (28) | USA | RCT | Mixed | 566 | 566 | CAM-ICU (once every 12 hours) | To examine the effects of haloperidol or ziprasidone on delirium during critical illness. | Low |
| Green, C. (2019) (29) | Australia | Prospective cohort study | Mixed | 455 | 160 | CAM-ICU (once every 12 hours) | To investigate the ability of available delirium risk assessment tools to identify patients at risk of delirium in an Australian tertiary ICU. | Unclear |
| Green, C. (2017) (30) | Scotland | Case-control study | Mixed | 30 | 15 | CAM-ICU (once every 12 hours) | To evaluate performance of a novel, graded test for objectively detecting inattention in delirium, implemented on a custom-built computerized device (Edinburgh Delirium Test Box-ICU). | Poor |
| Guenther, U (2013) (31) | Germany | Prospective cohort study | Cardiac | 215 | 69 | CAM-ICU (once a day) | To identify predisposing and precipitating risk factors of ICU delirium in a cardiosurgical, 12-bed ICU of a university hospital. | Good |
| Guenther, U. (2012) (32) | Germany | Prospective cohort study | Surgical | 160 | 62 | CAM-ICU (once a day) | To determine frequency of discrepancies between subjective and objective delirium monitoring. | Unclear |
| Guenther, U. (2010) (33) | Germany | Prospective cohort study | Surgical | 54 | 25 | DSM-IV + CAM-ICU (once a day) | To determine the validity and reliability of the CAM-ICU Flowsheet, a practical, time-sparing algorithm to assess the 4 delirium criteria in intubated patients | Unclear |
| Guenther, U. (2009) (34) | Germany | Prospective cohort study | Surgical | 82 | 35 | CAM-ICU (NA) | To test the CAM-ICU in comparison with clinical judgment alone, and the practicability of routine clinical delirium monitoring with the CAM-ICU. | Poor |
| Gusmao-Flores, D. (2011) (35) | Brazil | Prospective cohort study | Mixed | 119 | 46 | DSM-IV + CAM-ICU + ICDSC (two evaluations every week) | To compare the sensitivity and specificity of three diagnostic tools for delirium in a mixed population of critically ill patients, and to validate the Brazilian Portuguese CAM-ICU. | Poor |
| Haenggi, M. (2013) (36) | Switzerland | Prospective cohort study | Mixed | 80 | 58 | CAM-ICU + ICDSC (once a day) | To test the hypothesis that reduced arousability (RASS, scores -2 to -3) for any reason during delirium assessment increases the apparent prevalence of delirium in intensive care patients. | Poor |
| Helms, J. (2020) (37) | France | Prospective cohort study | Mixed | 140 | 97 | CAM-ICU (twice daily) | To describe the incidence of delirium AND/OR abnormal neuro- logical examination and compare the outcome of these patients to patients without delirium or any neurological symptoms. | Unclear |
| Herrera-Herrera, J. L. (2019) (38) | Colombia | Prospective cohort study | Mixed | 596 | 131 | CAM-ICU (NA) | To determine the incidence of delirium and related factors in patients admitted to an ICU of a health institution in the city of Monteria, Colombia. | Poor |
| Horacek, R. (2016) (39) | Czech Republic | Prospective cohort study | Surgical | 140 | 140 | Unclear (once every 6 hours) | To examine the impact of somatic illnesses, electrolyte imbalance, red blood cell count, hypotension, and antipsychotic and opioid treatment on the duration of delirium in Central ICU for Surgery. | Poor |
| Ishibashi-Kanno, N. (2020) (40) | Japan | Retrospective cohort study | Surgical | 69 | 23 | DSM-IV (NA) | To perform a statistical evaluation of the risk factors for postoperative delirium after oral tumor resection and reconstructive surgery. | Poor |
| Iwata, E. (2020) (41) | Japan | Prospective cohort study | Cardiac | 408 | 109 | CAM-ICU (once every 8 hours) | To elucidate whether delirium affects adverse outcomes of AHF, and to find risk factors that contribute to the development of delirium in patients with AHF treated in the ICU. |  |
| Jayaswal, A. K. (2019) (42) | India | Prospective cohort study | Medical | 280 | 88 | CAM-ICU (once every 12 hours) | To evaluate the incidence, motor subtypes, risk factors, and clinical outcome of delirium in the medical ICU. | Unclear |
| Jhala, N. (2015) (43) | USA | Prospective cohort study | Mixed | 100 | 30 | CAM-ICU (once every 12 hours) | To compare rates of detection of ICU delirium using current standard of care at St Vincent Charity Medical Center (SVCMC) to the standardized tool CAM-ICU and evaluates risk factors of delirium at SVCMC ICU. | Poor |
| Kanova, M. (2017) (44) | Czech Republic | Prospective cohort study | Mixed | 332 | 74 | CAM-ICU (once a day) | To evaluate the incidence of delirium and risk factors for delirium in a mixed group of trauma, medical and surgical ICU patients. | Poor |
| Karadas, C. (2016) (45) | Turkey | RCT | Medical | 94 | 14 | CAM-ICU (once every 12 hours) | To determine the effect of range of motion exercises on preventing delirium and shortening the duration of delirium among patients in the intensive care unit who are aged 65 and over. | High |
| Kawatani, Y. (2015) (46) | Japan | Retrospective cohort study | Surgical | 81 | 20 | ICDSC (once a day) | To examine the rate of delirium development in the ICU after EVAR, as well as the associated preoperative risk factors and effects on the lengths of ICU and hospital stays. | Poor |
| Kerber, K. (2020) (47) | USA | Retrospective cohort study | Medical | 171 | 23 | CAM-ICU (once every 12 hours) | To determine the occurrence rate of each delirium type in the first 7 days after intensive care unit admission and explore the relationship between delirium type and ventilatory outcomes. | Unclear |
| Khan, S. H. (2020) (48) | USA | Prospective cohort study | Mixed | 2742 | 452 | CAM-ICU (once every 12 hours) | To determine ICU delirium rates over time. | Poor |
| Khan, S. H. (2020) (49) | USA | Retrospective cohort study | Mixed | 268 | 215 | CAM-ICU (once every 12 hours) | To determine delirium occurrence rate, duration, and sever  ity in patients admitted to the ICU with coronavirus disease 2019. | Good |
| Klugkist, M. (2008) (50) | Germany | Prospective cohort study | Cardiac | 194 | 55 | CAM-ICU (once a day) | To answer if a German version of the CAM-ICU can be applied to patients after cardiac surgery if patients with and without the diagnosis of delirium differ in the clinical variables usually associated with this disorder in cardiac surgery. | Poor |
| Kumar, A. K. (2017) (51) | India | Prospective cohort study | Cardiac | 120 | 21 | CAM-ICU (once a day) | To prospectively identify the incidence, motoric subtypes, and risk factors associated with development of delirium in cardiac surgical patients admitted to postoperative cardiac intensive care, using a validated delirium monitoring instrument. | Poor |
| Lahariya, S. (2016) (52) | India | Prospective cohort study | Cardiac | 309 | 81 | DSM-IV-TR + DRS-R-98 + DMSS (once a day) | To study the phenomenology and motor sub-types of delirium in patients admitted in a CCU. | Unclear |
| Lee, A. (2018) (53) | China | Prospective cohort study | Cardiac | 600 | 83 | CAM-ICU (once every 8 hours) | To evaluate the association between motor subtypes of postoperative delirium in the intensive care unit and fast-track failure (a composite outcome of prolonged stay in the intensive care unit >48 hours, intensive care unit readmission, and 30-day mortality) after cardiac surgery. | Good |
| Lee, H. (2018) (54) | Korea | Retrospective cohort study | Surgical | 527 | 119 | CAM-ICU (once every 8 hours) | To evaluate the effect of timing and duration of delirium in patients who required ICU care for >24 hours after surgery and to evaluate the risk factors of POD in surgical ICU patients. | Unclear |
| Lee, H. J. (2018) (55) | Korea | Prospective cohort study | Neuro | 193 | 23 | CAM-ICU (NA) | To describe the incidence rate of delirium and to identify factors influencing delirium in neurological intensive care unit patients. | Good |
| Leite, M. A. (2014) (56) | Brazil | Prospective cohort study | Mixed | 64 | 34 | CAM-ICU (once every 12 hours) | To compare the incidence of delirium before and after extubation and to identify the risk factors and possible predictors for the occurrence of delirium. | Poor |
| Li, X. (2020) (57) | China | Prospective cohort study | Mixed | 115 | 76 | CAM-ICU (once every 12 hours) | To explore the incidence rate of delirium and to determine the risk factors among critically ill older patients. | Unclear |
| Lin, W. L. (2015) (58) | Taiwan | Prospective cohort study | Medical | 90 | 68 | CAM-ICU (once every 12 hours) | To establish the incidence rate of delirium among ICU elderly patients and to identify its risk factors. | Unclear |
| Lin, S. M. (2004) (59) | Taiwan | Prospective cohort study | Medical | 102 | 22 | DSM-IV + CAM-ICU (once a day) | To revalidate a means of assessing delirium in ICU patients and to investigate the independent effect of delirium on the mortality of mechanically ventilated patients. | Good |
| Linkaite, G. (2018) (60) | Lithuania | Prospective cohort study | Mixed | 38 | 22 | CAM-ICU + haloperidol administration (NA) | To validate the PRE-DELIRIC model using the CAM-ICU method for the diagnosis of delirium. | Poor |
| Maniar. H. S. (2016) (61) | USA | Retrospective cohort study | Cardiac | 427 | 135 | CAM-ICU (once every 12 hours) | To determine the incidence and clinical significance of POD in patients with aortic stenosis undergoing surgical aortic valve replacement (SAVR) or transcatheter aortic valve replacement (TAVR). | Unclear |
| McPherson, J. A. (2013) (62) | USA | Prospective cohort study | Cardiac | 200 | 53 | CAM-ICU (once a day) | To determine the prevalence and risk factors for delirium among cardiac surgery ICU patients. | Good |
| Mesa, P. (2017) (63) | Uruguay | Prospective cohort study | Mixed | 230 | 184 | DSM-V + CAM-ICU (daily in the morning shift and, in cases of agitation, during other shifts) | To establish the prevalence of delirium in a general ICU and to identify associated factors, clinical expression and the influence on outcomes. | Poor |
| Mitasova, A. (2012) (64) | Czech Republic | Prospective cohort study | Neuro | 129 | 55 | DSM-IV + CAM-ICU (once a day) | To describe the epidemiology and time spectrum of delirium using DSM-IV criteria and to validate a tool for delirium assessment in patients in the acute post stroke period. | Good |
| Nagari, N. (2019) (65) | India | Prospective cohort study | Medical | 1582 | 406 | CAM-ICU (NA) | To assess risk factors and precipitating factors of delirium in patients admitted to medical intensive care unit of a tertiary care hospital. | Poor |
| Needham, D. M. (2016) (66) | USA | RCT | Mixed | 272 | 195 | CAM-ICU (once a day) | To assess whether the pleiotropic effects of statins can reduce delirium in intensive care and decrease subsequent cognitive impairment in a randomized controlled trial. | Low |
| Nguyen, D. N. (2018) (67) | Belgium | Prospective cohort study | Mixed | 252 | 185 | CAM-ICU (once every 12 hours) | To investigate whether delirium would be more likely to develop in patients with low systemic arterial pressure, hypoxemia and a higher positive fluid balance in patients with shock. | Unclear |
| Nimali Lochanie, P. A. (2018) (68) | Sri Lanka | Prospective cohort study | Surgical | 30 | 20 | CAM-ICU (once a day) | To assess the incidence of and risk factors for delirium in mechanically ventilated patients in surgical ICU of National Hospital of Sri Lanka. | Poor |
| Nishimura, K. (2015) (69) | Japan | Prospective cohort study | Cardiac | 31 | 12 | DSM-IV-TR + CAM-ICU + ICDSC (only when all three evaluators were simultaneously present at the hospital) | To compare the CAM-ICU and ICDSC for detecting post-cardiac surgery delirium | Unclear |
| Oh, J. (2018) (70) | Korea | Prospective cohort study | Mixed | 94 | 39 | DSM-V + CAM-ICU + K-DRS + DMSS + delirium etiology checklist (once every 8 hours) | To investigate whether delirious patients can be successfully distinguished from non-delirious patients by using heart rate variability and machine learning. | Poor |
| Ozkul, A. (2019) (71) | Turkey | Case-control study | Mixed | 103 | 53 | DSM-IV + CAM-ICU (NA) | To investigate blood levels of the inflammatory mediator interleukin (IL)-6 and the neuron degeneration markers S100B and NSE in hyperactive, hypoactive, and mixed-delirium subtypes. | Poor |
| Ozturk, B. A. (2018) (72) | Turkey | Cross-sectional study | Cardiac | 130 | 6 | CAM-ICU (NA) | To evaluate the relationship of delirium and risk factors for cardiology ICU patients with the nursing workload. | Poor |
| Ozturk, B. A. (2017) (73) | Turkey | Quasi-experimental | Medical | 95 | 22 | CAM-ICU (once a day) | To investigate the effect of nonpharmacological intervention training on delirium recognition and the intervention strategies of ICU nurses. | Poor |
| Pan, A. (2019) (74) | China | Prospective cohort study | Mixed | 452 | 163 | CAM-ICU (once a day) | To investigate the incidence, risk factors, and cumulative risk of delirium among ICU patients. | Poor |
| Pandharipande, P. (2007) (75) | USA | Prospective cohort study | Mixed | 97 | 68 | CAM-ICU (once a day) | To identify the prevalence of the motoric subtypes of delirium in surgical and trauma ICU patients. | Poor |
| Park, S. (2020) (76) | Korea | Prospective cohort study | Surgical | 126 | 126 | CAM-ICU + DMSS-4 + DMC + DRS-98-R (once every 12 hours) | To investigate the motor subtypes of delirium in patients in a Surgical  Intensive Care Unit (SICU), and identify the factors related to the characteristics of patients according to the  motor subtypes of delirium. | Unclear |
| Peterson, J. (2006) (77) | USA | Prospective cohort study | Medical | 614 | 375 | CAM-ICU (once every 12 hours) | To describe the motoric subtypes of delirium in critically ill patients and compare patients aged 65 and older with a younger cohort | Good |
| Pipanmekaporn, T. (2015) (78) | Thailand | Prospective cohort study | Surgical | 4450 | 162 | ICDSC (once a day) | To determine the incidence and risk factors of delirium in Thai university-based surgical intensive care units. | Good |
| Plaschke, K. (2006) (79) | Germany | Prospective cohort study | Cardiac | 114 | 32 | CAM-ICU (once a day) | To investigate bilateral bispectral (BIS) index, cortisol as a stress marker, and interleukin-6 as a marker of inflammation in delirious patients after cardiac surgery. | Poor |
| Price, C. C. (2017) (80) | USA | Prospective cohort study | Cardiac | 594 | 137 | CAM-ICU (once every 12 hours) | To determine if certain cognitive domains from the general cognitive screener, the MMSE, predict delirium after cardiac surgery. | Unclear |
| Radtke, F. M. (2009) (81) | Germany | Prospective cohort study | Mixed | 68 | 47 | DSM-IV + ICDSC (NA) | To translate the ICDSC from English to German and to validate ICDSC according to the Guidelines of the Translation and Cultural Adaptation Group of Patient Reported Outcomes Measures - Principles of Good Practice | Unclear |
| Ritter, C. (2014) (82) | Brazil | Prospective cohort study | Mixed | 78 | 31 | CAM-ICU (once a day) | To study the relationship between systemic inflammation and the development of delirium in septic and non-septic critically ill patients. | Good |
| Robinson, D. (2019) (83) | USA | Prospective cohort study | Neuro | 55 | 19 | CAM-ICU (once a day) | To determine whether RASS scores could be used to reliably diagnose delirium in the setting of brain injury and to examine clinical factors associated with delirium in patients with subdural hematomas. | Good |
| Robinson, T. N. (2014) (84) | USA | RCT | Surgical | 301 | 116 | CAM-ICU (once a day) | To determine whether the postoperative administration of tryptophan would be beneficial for elderly adults undergoing surgery who are at risk of developing postoperative delirium. | Low |
| Robinson, T. N. (2011) (85) | USA | Prospective cohort study | Surgical | 172 | 74 | CAM-ICU (once a day) | To investigate the incidence, natural history, risk factors, outcomes and adverse events of the motor subtypes of POD | Good |
| Rood, P. J. T. (2019) (86) | The Netherlands | Retrospective cohort study | Mixed | 6323 | 1600 | CAM-ICU (once every 8 hours) | To determine the association between different delirium subtypes and 90-day mortality. | Unclear |
| Rosa, R. G. (2017) (87) | Brazil | Prospective Before-After study | Mixed | 286 | 43 | CAM-ICU (once every 12 hours) | To evaluate the effect of an extended visitation model compared with a restricted visitation model on the occurrence of delirium among ICU patients. | Good |
| Rudiger, A. (2016) (88) | Switzerland | Retrospective cohort study | Cardiac | 194 | 50 | CAM-ICU + ICDSC (once every 8 hours) | To investigate whether intra-operative pathophysiological alterations and therapeutic interventions influence the risk of POD. | Unclear |
| Saida, I, B, (2020) (89) | Tunesia | Retrospective cohort study | Medical | 10 | 5 | CAM-ICU (NA) | To describe clinical features and outcomes of critically ill patients with SARS-CoV-2 infection | Poor |
| Sanchez-Hurtado, L. A. (2018) (90) | Mexico | Prospective cohort study | Medical | 109 | 25 | CAM-ICU (once a day) | To estimate the incidence of delirium and its risk factors among critically ill cancer patients in an ICU | Poor |
| Sanson, G. (2018) (91) | Italy | Prospective cohort study | Cardiac | 199 | 61 | ICDSC (once every 8 hours) | To evaluate risk factors, incidence, fluctuations, phenotypic characteristics and impact on patients' outcomes of delirium. | Good |
| Shadvar, K. (2013) (92) | Iran | Prospective cohort study | Cardiac | 200 | 47 | CAM-ICU (NA) | To evaluate the prevalence and risk factors of delirium in patients admitted to cardiac surgery. | Unclear |
| Sharma, A. (2012) (93) | India | Prospective cohort study | Mixed | 140 | 75 | DSM-IV + DRS-R-98 (once a day) | To evaluate the incidence, prevalence, risk factors and outcome of delirium in the respiratory ICU of a tertiary care hospital. | Good |
| Shaughnessy, L. (2013) (94) | United Kingdom | Prospective cohort study | Cardiac | 108 | 23 | CAM-ICU (once every 8 hours) | To share an experience of introducing delirium scoring into a Cardiothoracic Critical Care Unit and the lessons learnt. | Poor |
| Simeone, S. (2018) (95) | Italy | Prospective cohort study | Cardiac | 89 | 65 | CAM-ICU (NA) | To observe the clinical and structural factors that can be associated with the post-operative onset of delirium in patients who have undergone heart surgery. | Poor |
| Simons, K. S. (2018) (96) | The Netherlands | Prospective cohort study | Mixed | 50 | 35 | CAM-ICU (once every 8 hours) | To investigate whether or not serial determinations of markers may improve this association. | Poor |
| Smit, L. (2021) (97) | The Netherlands | Prospective cohort study | Mixed | 3614 | 1165 | A delirium recognition algorithm based on CAM-ICU + chart review + additional delirium assessments by trained researchers (once a day) | To determine whether haloperidol, clonidine, or their combined administration to delirious ICU patients results in delirium resolution. | Good |
| Smulter, N (2013) (98) | Sweden | Prospective cohort study | Cardiac | 142 | 78 | DSM-IV-TR (preoperatively and on Day 1 and Day 4 postoperatively) | To explore risk factors behind delirium in older patients undergoing cardiac surgery with cardiopulmonary bypass. | Poor |
| Soehle, M. (2015) (99) | Germany | Prospective cohort study | Cardiac | 81 | 26 | CAM-ICU (once a day) | To analyze whether perioperative bilateral BIS monitoring may detect abnormalities before the onset of POD in cardiac surgery patients. | Poor |
| Stransky, M. (2011) (100) | Germany | Prospective cohort study | Cardiac | 467 | 54 | CAM-ICU + ICDSC (once a day) | To evaluate the incidence of the three subtypes of delirium, the risk factors of the subtypes in cardiac surgery, and the impact of the subtypes on clinical outcomes. | Poor |
| Su, X. (2016) (101) | China | RCT | Surgical | 700 | 86 | CAM-ICU (once every 12 hours) | To investigate whether prophylactic low-dose dexmedetomidine, could safely decrease the incidence of delirium in elderly patients after non-cardiac surgery. | Low |
| Sun, L. (2016) (102) | China | Case-control study | Surgical | 112 | 56 | CAM-ICU (once every 12 hours) | To elucidate the pathogenesis of POD in elderly oral cancer patients by determining the perioperative kinetics of inflammatory cytokines, cortisol, and amyloid β1-40. | Poor |
| Svenningsen, H. (2013) (103) | Denmark | Prospective cohort study | Mixed | 640 | 419 | CAM-ICU (once every 12 hours) | To investigate the impact of fluctuating sedation levels on the incidence of delirium in ICU. | Good |
| Svenningsen, H. (2011) (104) | Denmark | Prospective cohort study | Mixed | 102 | 41 | CAM-ICU (once every 12 hours) | To evaluate the incidence of delirium in adult ICU patients in Denmark and to identify correlations between delirium, sedatives, opioid analgesics and age. | Poor |
| Szwed, K. (2021) (105) | Poland | RCT | Cardiac | 191 | 52 | CAM-ICU (once every 12 hours) | To determine whether using any of the 2 selected modifications of OPCAB could decrease the incidence of these undesired sequelae. | Unclear |
| Tate, J. A. (2013) (106) | USA | Quasi-experimental sequential cohort study | Mixed | 89 | 38 | CAM-ICU (NA) | To investigate the prevalence of delirium and delirium subtypes in a sample of intubated ICU patients across 5 time-points. | Poor |
| Thom, R. P. (2018) (107) | USA | Retrospective cohort study | Medical | 322 | 322 | CAM-ICU (once every 8 hours) | To investigate the effect of early versus late versus no antipsychotic administration on ICU delirium. | Poor |
| Tilouche, N. (2018) (108) | Tunesia | Prospective cohort study | Medical | 206 | 39 | CAM-ICU (once a day) | To determine the incidence, risk factors, and impact on outcome of delirium in a medical ICU in Tunisia. | Poor |
| Tobar, E. (2010) (109) | Chile | Prospective cohort study | Mixed | 29 | 18 | DSM-IV-TR + CAM-ICU (once a day) | To adapt the CAM-ICU for the diagnosis of delirium to the language and culture of Spain and to validate the adapted version | Poor |
| Torres-Contreras, C. (2019) (110) | Colombia | Prospective cohort study | Mixed | 134 | 27 | CAM-ICU (NA) | To determine the incidence and the factors associated with delirium in intensive care unit patients. | Poor |
| Tsuruta, R (2010) (111) | Japan | Prospective cohort study | Mixed | 103 | 21 | CAM-ICU (once a day) | To investigate the prevalence and associated factors of delirium in critically ill patients during an ICU stay. | Poor |
| Uguz, F. (2010) (112) | Turkey | Prospective cohort study | Cardiac | 212 | 12 | DSM-IV-TR (once a day) | To examine the incidence, clinical profile, and predictors of delirium following acute myocardial infarction. | Poor |
| Van Dellen, E. (2014) (113) | The Netherlands | Case-control study | Cardiac | 49 | 25 | CAM-ICU (once a day) | To improve the understanding of the pathophysiology and phenomenology of delirium by exploring functional connectivity and network topology in electroencephalography recordings of patients with delirium after cardiac surgery. | Unclear |
| Van den Boogaard, M. (2013) (114) | Netherlands | Prospective Before-After study | Mixed | 476 | 340 | CAM-ICU (once every 8 hours) | To evaluate if our changed delirium policy resulted in quality improvement of relevant delirium outcome measures. | Poor |
| Van den Boogaard, M. (2012) (115) | The Netherlands | Prospective Cohort study | Mixed | 1613 | 411 | CAM-ICU (once every 8 hours) | To determine the overall incidence and duration of delirium, per delirium subtype and per ICU admission diagnosis. Furthermore, we determined the short-term consequences of delirium. | Good |
| Van der Kooi, A. (2015) (116) | The Netherlands | Case-control study | Cardiac | 56 | 28 | CAM-ICU (NA) | To determine the electrode derivation and EEG characteristic that have the best capability to distinguish patients with delirium from patients without delirium. | Unclear |
| Van Eijk, M. M. (2011) (117) | The Netherlands | Prospective cohort study | Mixed | 282 | 80 | DSM-IV + CAM-ICU (within 3 hours of the expert assessment, the day before and the day after the experts visits) | To investigate the diagnostic value of the CAM-ICU in daily practice. | Poor |
| Van Eijk, M. M. (2009) (118) | The Netherlands | Prospective cohort study | Mixed | 126 | 43 | DSM-IV, CAM-ICU, ICDSC (NA) | To compare the value of three detection methods (CAM-ICU, ICDSC) and the impression of the ICU physician with the diagnosis of a psychiatrist, neurologist, or geriatrician. | Unclear |
| Van Ewijk, C. E. (2016) (119) | Netherlands | Prospective cohort study | Mixed | 61 | 61 | CAM-ICU + haloperidol administration (once every 8 hours) | To determine the number of patients admitted to the ICU or MCU who are diagnosed with delirium and who show characteristics of serotonin toxicity in association with the administration of serotonergic drugs. | Unclear |
| Van Keulen, K. (2018) (120) | The Netherlands | Prospective cohort study | Mixed | 410 | 410 | CAM-ICU (once a day) | To determine whether estimates of glucose variability are altered during delirium in critically ill patients with and without diabetes in the ICU. | Poor |
| Voils, S. A. (2020) (121) | USA | Case-control study | Surgical | 130 | 65 | CAM-ICU (once every 12 hours) | To determine metabolomic signatures associated with delirium in critically ill and injured patients. | Unclear |
| Vyveganathan, L. (2019) (122) | Malaysia | Prospective cohort study | Mixed | 139 | 59 | CAM-ICU (once a day) | To determine the incidence and evaluate the risk factors and outcomes of delirium in general ICU. | Unclear |
| Wang, C. (2013) (123) | China | Prospective cohort study | Mixed | 126 | 61 | DSM-IV + CAM-ICU (once every 12 hours) | To provide a method for delirium evaluation in simplified Chinese for patients speaking this language via validation of a translation of the CAM-ICU. | Unclear |
| Wang, J. (2020) (124) | China | Prospective cohort study | Neuro | 310 | 118 | CAM-ICU (once a day) | To establish a delirium prediction model for neurosurgical ICU patients and to verify the sensitivity and specificity of this model. | Good |
| Wang, J. (2018) (125) | China | Prospective cohort study | Neuro | 128 | 54 | CAM-ICU (once every 12 hours) | To explore the incidence of delirium in cerebrovascular patients admitted in our Neurosurgery ICU and analyze the risk factors leading to delirium. | Poor |
| Whitlock, E. L. (2014) (126) | USA | RCT | Cardiac | 310 | 73 | CAM-ICU (once every 12 hours) | To determine whether there was a difference in postoperative delirium between patients randomized to BIS-guided or end-tidal anesthetic concentration (ETAC)-guided protocols. | Low |
| Woien, H. (2013) (127) | Norway | Prospective cohort study | Mixed | 139 | 32 | CAM-ICU (once every 8 hours) | To test the usefulness of the CAM-ICU and to describe the incidence of delirium in critically ill patients at two Norwegian hospitals. | Good |
| Yasayacak, A (2012) (128) | Turkey | Prospective cohort study | Cardiac | 55 | 10 | CAM-ICU (NA) | To determine delirium and risk factors in patients in cardiovascular surgery ICU. | Unclear |
| Zhang, W. (2017) (129) | China | Prospective Before-After Study | Cardiac | 278 | 60 | CAM-ICU (once every 8 hours) | To determine whether a nursing intervention targeting risk factors could decrease the incidence of POD among patients who had coronary artery bypass grafting in China. | Unclear |
| Zhang, W. (2016) (130) | China | Prospective cohort study | Cardiac | 95 | 103 | CAM-ICU (once every 8 hours) | To explore the profiles of delirium in coronary artery bypass grafting patients and identify related patient outcomes. | Poor |
| Zujalovic, B. (2020) (131) | Germany | Prospective cohort study | Surgical | 175 | 76 | CAM-ICU (once every 8 hours) | To test whether a longitudinal analysis of acetylcholinesterase activity in critically ill patients can help to diagnose a suspected septic-associated encephalopathy and whether acetylcholinesterase-activity differs in comparison to non-septic patients. | Unclear |

ICU = Intensive care unit, SDU = Step down unit, MCU = Medium care unit, CCU = Coronary care unit, DSM–III–R = Diagnostic and Statistical Manual, 3rd edition, revised, DMS-IV = Diagnostic and Statistical Manual, 4th edition, DSM-IV-TR = Diagnostic and Statistical Manual, 4th edition, text revision, DSM-V = Diagnostic and Statistical Manual, 5th edition, CAM = Confusion Assessment Method, CAM-ICU = Confusion Assessment Method for the Intensive Care Unit, ICDSC = Intensive Care Delirium Screening Checklist, ICD-9 = International Classification of Diseases, 9th Revision, ICD-9-CM = International Classification of Diseases, 9th Revision, Clinical Modification, ICD-10 = International Classification of Diseases, 10th Revision, DMSS = Delirium Motor Subtype Scale, MMSE = Mini-Mental State Exam, PAD = Pain, agitation, and delirium, POD = Postoperative delirium, RASS = Richmond Agitation Sedation Scale

*Cochrane RoB with allocation of either high, unclear or low risk of bias was used for RCTs and NIH quality assessment tools with allocation of either poor, unclear or good quality were used for observational studies

**Reference list for e-Table 1**

1. Agarwal V, O’Neill PJ, Cotton BA, Pun BT, Haney S, Thompson J, et al. Prevalence and risk factors for development of delirium in burn intensive care unit patients. J Burn Care Res. 2010;31(5):706–15.

2. Aldemir M, Ozen S, Kara IH, Sir A, Bac B. Predisposing factors for delirium in the surgical intensive care unit. Crit Care. 2001;5(5):265–70.

3. Angles EM, Robinson TN, Biffl WL, Johnson J, Moss M, Tran Z V, et al. Risk factors for delirium after major trauma. Am J Surg. 2008;196(6):864–70.

4. Anlacan, Veeda Michelle M.; Tuble, Genevieve C.; Saranza, Gerard M.; Albay, Albert B.; Sumalapao DEP. Prevalence of delirium in patients admitted at intensive care units of Philippine General Hospital. Phil J Chest Dis. 2015;16(03):27–33.

5. Arroyo-Novoa CM, Figueroa-Ramos MI, Puntillo KA. Occurrence and Practices for Pain, Agitation, and Delirium in Intensive Care Unit Patients. P R Health Sci J. 2019;38(3):156–62.

6. Artemiou P, Bily B, Bilecova-Rabajdova M, Sabol F, Torok P, Kolarcik P, et al. Melatonin treatment in the prevention of postoperative delirium in cardiac surgery patients. Kardiochirurgia i Torakochirurgia Pol. 2015;12(2):126–33.

7. Ayazoglu TA, Baysal A, Tur H, Ozkaynak I. Prevalence and risk factors for postoperative delirium in a cardiac surgery Intensive Care Unit. J Exp Clin Med. 2012;29(2):101–7.

8. Barman A, Pradhan D, Bhattacharyya P, Dey S, Bhattacharjee A, Tesia SS, et al. Diagnostic accuracy of delirium assessment methods in critical care patients. J Crit Care. 2018;44:82–6.

9. Bigatello LM, Amirfarzan H, Haghighi AK, Newhouse B, Del Rio JM, Allen K, et al. Effects of routine monitoring of delirium in a surgical/trauma intensive care unit. J Trauma Acute Care Surg. 2013;74(3):876–83.

10. Soenke B, Garcia ND, Rafael M, André R, Alain R, Maria S, et al. Screening for delirium with the Intensive Care Delirium Screening Checklist (ICDSC): A re-evaluation of the threshold for delirium. Swiss Med Wkly. 2018;

11. Breu A, Stransky M, Metterlein T, Werner T, Trabold B. Subsyndromal delirium after cardiac surgery. Scand Cardiovasc J. 2015;49(4):207–12.

12. Brown CH, Laflam A, Max L, Lymar D, Neufeld KJ, Tian J, et al. The Impact of Delirium after Cardiac Surgical Procedures on Postoperative Resource Use. Ann Thorac Surg. 2016;101(5):1663–9.

13. Bui LN, Pham VP, Shirkey BA, Swan JT. Effect of delirium motoric subtypes on administrative documentation of delirium in the surgical intensive care unit. J Clin Monit Comput. 2017;31(3):631–40.

14. Canet E, Amjad S, Robbins R, Lewis J, Matalanis M, Jones D, et al. Differential clinical characteristics, management and outcome of delirium among ward compared with intensive care unit patients. Intern Med J. 2019;49(12):1496–504.

15. Caruso P, Guardian L, Tiengo T, Dos Santos LS, Junior PM. ICU architectural design affects the delirium prevalence: a comparison between single-bed and multibed rooms*. Crit Care Med. 2014;42(10):2204–10.

16. Castañoa, Ángela María Henao; Amaya-Reyb MCDP. Delirium in awake patients with mechanical ventilation in intensive care unit. Rev Latinoam Bioética. 2015;15(1):120–9.

17. Ceriana P, Fanfulla F, Mazzacane F, Santoro C, Nava S, Cerianna P, et al. Delirium in patients admitted to a step-down unit: Analysis of incidence and risk factors. J Crit Care. 2010;25(1):136–43.

18. Chanidnuan M, Pancharoen W, Toadithep P, Chaiwat O, Thanakiattiwibun C, Vijitmala K, et al. Postoperative delirium in critically ill surgical patients: incidence, risk factors, and predictive scores. BMC Anesthesiol. 2019;19(1):39.

19. Collet MO, Caballero J, Sonneville R, Bozza FA, Nydahl P, Schandl A, et al. Prevalence and risk factors related to haloperidol use for delirium in adult intensive care patients: the multinational AID-ICU inception cohort study. Intensive Care Med. 2018;44(7):1081–9.

20. de la Varga-Martínez O, Gómez-Pesquera E, Muñoz-Moreno MF, Marcos-Vidal JM, López-Gómez A, Rodenas-Gómez F, et al. Development and validation of a delirium risk prediction preoperative model for cardiac surgery patients (DELIPRECAS): An observational multicentre study. J Clin Anesth. 2021;69:110158.

21. Devlin JW, Skrobik Y, Riker RR, Hinderleider E, Roberts RJ, Fong JJ, et al. Impact of quetiapine on resolution of individual delirium symptoms in critically ill patients with delirium: a post-hoc analysis of a double-blind, randomized, placebo-controlled study. Crit Care. 2011;15(5):R215.

22. Eremenko AA, Chernova E V. [Treatment of delirium in the early postoperative period after cardiac surgery]. Anesteziol Reanimatol. 2014;30–4.

23. Estrup S, Kjer CKW, Poulsen LM, Gøgenur I, Mathiesen O. Delirium and effect of circadian light in the intensive care unit: a retrospective cohort study. Acta Anaesthesiol Scand. 2018;62(3):367–75.

24. Falsini G, Grotti S, Porto I, Toccafondi G, Fraticelli A, Angioli P, et al. Long-term prognostic value of delirium in elderly patients with acute cardiac diseases admitted to two cardiac intensive care units: a prospective study (DELIRIUM CORDIS). Eur Hear journal Acute Cardiovasc care. 2018;7(7):661–70.

25. Fritz BA, Kalarickal PL, Maybrier HR, Muench MR, Dearth D, Chen Y, et al. Intraoperative Electroencephalogram Suppression Predicts Postoperative Delirium. Anesth Analg. 2016;122(1):234–42.

26. Garcia Nuñez D, Boettger S, Meyer R, Richter A, Schubert M, Meagher D, et al. Validation and Psychometric Properties of the German Version of the Delirium Motor Subtype Scale (DMSS). Assessment. 2019;26(8):1573–81.

27. Gaspardo P, Peressoni L, Comisso I, Mistraletti G, Ely EW, Morandi A. Delirium among critically ill adults: evaluation of the psychometric properties of the Italian “Confusion Assessment Method for the Intensive Care Unit.” Intensive Crit Care Nurs. 2014;30(5):283–91.

28. Girard TD, Exline MC, Carson SS, Hough CL, Rock P, Gong MN, et al. Haloperidol and Ziprasidone for Treatment of Delirium in Critical Illness. N Engl J Med. 2018;

29. Green C, Bonavia W, Toh C, Tiruvoipati R. Prediction of ICU Delirium: Validation of Current Delirium Predictive Models in Routine Clinical Practice. Crit Care Med. 2019;47(3):428–35.

30. Green C, Hendry K, Allerhand M, MacLullich AMJ, Tieges Z, Wilson ES, et al. A Novel Computerized Test for Detecting and Monitoring Visual Attentional Deficits and Delirium in the ICU. Crit Care Med. 2017;45(7):1224–31.

31. Guenther U, Theuerkauf N, Frommann I, Brimmers K, Malik R, Stori S, et al. Predisposing and precipitating factors of delirium after cardiac surgery: A prospective observational cohort study. Ann Surg. 2013;257(6):1160–7.

32. Guenther U, Weykam J, Andorfer U, Theuerkauf N, Popp J, Ely EW, et al. Implications of objective vs subjective delirium assessment in surgical intensive care patients. Am J Crit Care. 2012;21(1):e12–20.

33. Guenther U, Popp J, Koecher L, Muders T, Wrigge H, Ely EW, et al. Validity and reliability of the CAM-ICU flowsheet to diagnose delirium in surgical ICU patients. J Crit Care. 2010;25(1):144–51.

34. Gunther U, Wrigge H, Popp J, Andorfer U, Muders T, Putensen C. German short version of the confusion assessment method for the intensive care unit for routine delirium monitoring. [German]. Anasthesiol und Intensivmed. 2009;50(SUPPL. 5):592–600.

35. Gusmao-Flores D, Salluh JIF, Dal-Pizzol F, Ritter C, Tomasi CD, de Lima MASD, et al. The validity and reliability of the Portuguese versions of three tools used to diagnose delirium in critically ill patients. Clinics. 2011;66(11):1917–22.

36. Haenggi M, Blum S, Brechbuehl R, Brunello A, Jakob SM, Takala J. Effect of sedation level on the prevalence of delirium when assessed with CAM-ICU and ICDSC. Intensive Care Med. 2013;39(12):2171–9.

37. Helms J, Kremer S, Merdji H, Schenck M, Severac F, Clere-Jehl R, et al. Delirium and encephalopathy in severe COVID-19: A cohort analysis of ICU patients. Crit Care. 2020;24:491.

38. Herrera Herrera JL, Oyola Lopez E, Llorente Perez YJ. Delirium in patients of the Intensive Care Unit of a health institution in Monteria, Colombia. Rev Cient la Soc Esp Enferm Neurol. 2019;51:7–12.

39. Horacek R, Krnacova B, Prasko J, Latalova K. Delirium as a complication of the surgical intensive care. Neuropsychiatr Dis Treat. 2016;12:2425–34.

40. Ishibashi-Kanno N, Takaoka S, Nagai H, Okubo-Sato M, Fukuzawa S, Uchida F, et al. Postoperative delirium after reconstructive surgery for oral tumor: a retrospective clinical study. Int J Oral Maxillofac Surgery. 2020;49(9):1143–8.

41. Iwata E, Kondo T, Kato T, Okumura T, Nishiyama I, Kazama S, et al. Prognostic Value of Delirium in Patients With Acute Heart Failure in the Intensive Care Unit. Can J Cardiol. 2020;36(10):1649–57.

42. Jayaswal A, Sampath H, Soohinda G, Dutta S. Delirium in medical intensive care units: Incidence, subtypes, risk factors, and outcome. Indian J Psychiatry. 2019;61(4):352–8.

43. Jhala N, Babych I, Merugu S, Altaqi B. An observational study for detection of intensive care unit (ICU) delirium and delirium subtypes at St. Vincent charity medical center (SVCMC). St Vincent Charity Med J. 2015;1(5):12–7.

44. Kanova M, Sklienka P, Kula R, Burda M, Janoutova J. Incidence and risk factors for delirium development in ICU patients – A prospective observational study. Biomed Pap. 2017;161(2):187–96.

45. Karadas C, Ozdemir L. The effect of range of motion exercises on delirium prevention among patients aged 65 and over in intensive care units. Geriatr Nurs (Minneap). 2016;37(3):180–5.

46. Kawatani Y, Nakamura Y, Hayashi Y, Taneichi T, Ito Y, Kurobe H, et al. Development of delirium in the intensive care unit in patients after endovascular aortic repair: A retrospective evaluation of the prevalence and risk factors. Crit Care Res Pract. 2015;405817.

47. Kerber K, Zangmeister J, McNett M. Relationship Between Delirium and Ventilatory Outcomes in the Medical Intensive Care Unit. Crit Care Nurse. 2020;40(2):24–31.

48. Khan SH, Lindroth H, Hendrie K, Wang S, Imran S, Perkins AJ, et al. Time trends of delirium rates in the intensive care unit. Hear Lung. 2020;49:572–7.

49. Khan SH, Lindroth H, Perkins AJ, Jamil Y, Wang S, Roberts S, et al. Delirium Incidence, Duration, and Severity in Critically Ill Patients With Coronavirus Disease 2019. Crit Care Explor. 2020;2(12):p e0290.

50. Klugkist M, Sedemund-Adib B, Schmidtke C, Schmucker P, Sievers HH, Hüppe M, et al. Confusion Assessment Method for the Intensive Care Unit (CAM-ICU): diagnostik des postoperativen delirs bei kardiochirurgischen Patienten. Anaesthesist. 2008;57(5):464–74.

51. Kumar AK, Jayant A, Arya VK, Magoon R, Sharma R, Arya VK. Delirium after cardiac surgery: A pilot study from a single tertiary referral center. Ann Card Anaesth. 2017;20(1):76–82.

52. Lahariya S, Grover S, Bagga S, Sharma A. Phenomenology of delirium among patients admitted to a coronary care unit. Nord J Psychiatry. 2016;70(8):626–32.

53. Lee A, Mu JL, Chiu CH, Gin T, Underwood MJ, Joynt GM. Effect of motor subtypes of delirium in the intensive care unit on fast-track failure after cardiac surgery. J Thorac Cardiovasc Surg. 2018;155(1):268-275.e1.

54. Lee H, Ju JW, Oh SY, Kim J, Jung CW, Ryu HG. Impact of timing and duration of postoperative delirium: a retrospective observational study. Surg (United States). 2018;164(1):137–43.

55. Lee HJ, Kim SR. Factors Influencing Delirium in Neurological Intensive Care Unit Patient. Korean J Adult Nurs. 2018;30(5):470–81.

56. Leite MA, Osaku EF, Costa CRL de M, Cândia MF, Toccolini B, Covatti C, et al. Delirium during Weaning from Mechanical Ventilation. Crit Care Res Pract. 2014;546349.

57. Li X, Zhang L, Gong F, Ai Y. Incidence and Risk Factors for Delirium in Older Patients following Intensive Care Unit Admission: A Prospective Observational Study. J Nurs Res. 2020;28(4):p e101.

58. Lin WL, Chen YF, Wang J. Factors Associated with the Development of Delirium in Elderly Patients in Intensive Care Units. J Nurs Res. 2015;23(4):322–9.

59. Lin S, Liu C, Wang C, Lin H, Huang C, Huang P, et al. The impact of delirium on the survival of mechanically ventilated patients. Crit Care Med. 2004;32(11):2254–9.

60. Linkaitė G, Riauka M, Bunevičiūtė I, Vosylius S. Evaluation of PRE-DELIRIC (PREdiction of DELIRium in ICu patients) delirium prediction model for the patients in the intensive care unit. Acta medica Litu. 2018;

61. Maniar HS, Lindman BR, Escallier K, Avidan M, Novak E, Melby SJ, et al. Delirium after surgical and transcatheter aortic valve replacement is associated with increased mortality. J Thorac Cardiovasc Surg. 2016;151(3):815–23.

62. McPherson JA, Wagner CE, Boehm LM, Hall JD, Johnson DC, Miller LR, et al. Delirium in the cardiovascular ICU: Exploring modifiable risk factors. Crit Care Med. 2013;41(2):405–13.

63. Mesa P, Previgliano IJ, Altez S, Favretto S, Orellano M, Lecor C, et al. Delirium in a Latin American intensive care unit. A prospective cohort study of mechanically ventilated patients. Rev Bras Ter Intensiva. 2017/10/19. 2017;29(3):337–45.

64. Mitasova A, Kostalova M, Bednarik J, Michalcakova R, Kasparek T, Balabanova P, et al. Poststroke delirium incidence and outcomes: validation of the Confusion Assessment Method for the Intensive Care Unit (CAM-ICU). Crit Care Med. 2012;40(2):484–90.

65. Nagari N, Suresh Babu M. Assessment of risk factors and precipitating factors of delirium in patients admitted to intensive care unit of a tertiary care hospital. Br J Med Pract. 2019;12(2):a011.

66. Needham DM, Colantuoni E, Dinglas VD, Hough CL, Wozniak AW, Jackson JC, et al. Rosuvastatin versus placebo for delirium in intensive care and subsequent cognitive impairment in patients with sepsis-associated acute respiratory distress syndrome: An ancillary study to a randomised controlled trial. Lancet Respir Med. 2016;4(3):203–12.

67. Nguyen DN, Huyghens L, Parra J, Schiettecatte J, Smitz J, Vincent JL. Hypotension and a positive fluid balance are associated with delirium in patients with shock. PLoS One. 2018;13(8):e0200495.

68. Nimali Lochanie PA, Ranawaka NMD. Assessment of incidence and risk factors for intensive care acquired delirium in mechanically ventilated patients in surgical intensive care unit - National Hospital of Sri Lanka. Sri Lankan J Anaesthesiol. 2018;26(2):131–6.

69. Nishimura K, Yokoyama K, Yamauchi N, Koizumi M, Harasawa N, Yasuda T, et al. Sensitivity and specificity of the Confusion Assessment Method for the Intensive Care Unit (CAM-ICU) and the Intensive Care Delirium Screening Checklist (ICDSC) for detecting post-cardiac surgery delirium: A single-center study in Japan. Hear Lung J Acute Crit Care. 2016;45(1):15–20.

70. Oh J, Cho D, Park J, Na SH, Kim J, Heo J, et al. Prediction and early detection of delirium in the intensive care unit by using heart rate variability and machine learning. Physiol Meas. 2018;39(3):35004.

71. Ozkul A, Yilmaz A, Akyol A, Kaan N, Yenisey C, Ceylan A, et al. Biomarkers of neuronal damage and inflammation in delirium. Neurol Sci Neurophysiol. 2019;36(4):193–7.

72. Ozturk Birge A, Beduk T. The relationship of delirium and risk factors for cardiology intensive care unit patients with the nursing workload. J Clin Nurs. 2018;27(9–10):2109–19.

73. Ozturk Birge A, Tel Aydin H, Öztürk Birge A, Tel Aydin H. The effect of nonpharmacological training on delirium identification and intervention strategies of intensive care nurses. Intensive Crit Care Nurs. 2017;41:33–42.

74. Pan Y, Yan J, Jiang Z, Luo J, Zhang J, Yang K. Incidence, risk factors, and cumulative risk of delirium among ICU patients: A case-control study. Int J Nurs Sci. 2019;6(3):247–51.

75. Pandharipande P, Cotton BA, Shintani A, Thompson J, Costabile S, Truman Pun B, et al. Motoric subtypes of delirium in mechanically ventilated surgical and trauma intensive care unit patients. Intensive Care Med. 2007;33(10):1726–31.

76. Park SH. Factors Related to Motor Subtypes of Delirium Patients in a Surgical Intensive Care Unit. J Korean Clin Nurs Res. 2020;26(2):207–2016.

77. Peterson JF, Pun BT, Dittus RS, Thomason JWW, Jackson JC, Shintani AK, et al. Delirium and its motoric subtypes: a study of 614 critically ill patients. J Am Geriatr Soc. 2006;54(3):479–84.

78. Pipanmekaporn T, Chittawatanarat K, Chaiwat O, Thawitsri T, Wacharasint P, Kongsayreepong S, et al. Incidence and risk factors of delirium in multi-center Thai surgical intensive care units: A prospective cohort study. J Intensive Care. 2015;3(1):53.

79. Plaschke K, Fichtenkamm P, Schramm C, Hauth S, Martin E, Verch M, et al. Early postoperative delirium after open-heart cardiac surgery is associated with decreased bispectral EEG and increased cortisol and interleukin-6. Intensive Care Med. 2010;36(12):2081–9.

80. Price CC, Garvan C, Hizel LP, Lopez MG, Billings Iv FT, Hizel L, et al. Delayed Recall and Working Memory MMSE Domains Predict Delirium following Cardiac Surgery. J Alzheimer’s Dis. 2017;59(3):1027–35.

81. Radtke F, Franck M, Oppermann S, Lütz A, Seeling M, Heymann A, et al. Die Intensive Care Delirium Screening Checklist (ICDSC) – Richtlinienkonforme Übersetzung und Validierung einer intensivmedizinischen Delirium–Checkliste. AINS - Anästhesiologie · Intensivmed · Notfallmedizin · Schmerztherapie. 2009;44(2):80–6.

82. Ritter C, Tomasi CD, Dal-Pizzol F, Pinto BB, Dyson A, de Miranda AS, et al. Inflammation biomarkers and delirium in critically ill patients. Crit Care. 2014;23:R106.

83. Robinson D, Thompson S, Bauerschmidt A, Melmed K, Couch C, Park S, et al. Dispersion in Scores on the Richmond Agitation and Sedation Scale as a Measure of Delirium in Patients with Subdural Hematomas. Neurocrit Care. 2019;30(3):626–34.

84. Robinson TN, Dunn CL, Adams JC, Hawkins CL, Tran Z V, Raeburn CD, et al. Tryptophan Supplementation and Postoperative Delirium-A Randomized Controlled Trial. J Am Geriatr Soc. 2014;62(9):1764–71.

85. Robinson TN, Raeburn CD, Tran Z V., Brenner LA, Moss M. Motor subtypes of postoperative delirium in older adults. Arch Surg. 2011;146(3):295–300.

86. Rood PJT, van de Schoor F, van Tertholen K, Pickkers P, van den Boogaard M. Differences in 90-day mortality of delirium subtypes in the intensive care unit: A retrospective cohort study. J Crit Care. 2019;53:120–4.

87. Rosa RG, Tonietto TF, da Silva DB, Gutierres FA, Ascoli AM, Madeira LC, et al. Effectiveness and Safety of an Extended ICU Visitation Model for Delirium Prevention: A Before and After Study. Crit Care Med. 2017;45(10):1660–7.

88. Rudiger A, Begdeda H, Babic D, Krüger B, Seifert B, Schubert M, et al. Intra-operative events during cardiac surgery are risk factors for the development of delirium in the ICU. Crit Care. 2016;20:1–8.

89. Saida I Ben, Ennouri E, Nachi R, Meddeb K, Mahmoud J, Thabet N, et al. Very severe covid-19 in the critically ill in tunisia. Pan Afr Med J. 2020;35(suppl):136.

90. Sánchez-Hurtado LA, Hernández-Sánchez N, Del Moral-Armengol M, Guevara-García H, García-Guillén FJ, Herrera-Gómez Á, et al. Incidence of Delirium in Critically Ill Cancer Patients. Pain Res Manag. 2018;e4193275.

91. Sanson G, Khlopenyuk Y, Milocco S, Sartori M, Dreas L, Fabiani A. Delirium after cardiac surgery. Incidence, phenotypes, predisposing and precipitating risk factors, and effects. Hear Lung. 2018;47(4):408–17.

92. Shadvar K, Baastani F, Mahmoodpoor A, Bilehjani E. Evaluation of the Prevalence and Risk Factors of Delirium in Cardiac Surgery ICU. J Cardiovasc Thorac Res. 2013;5(4):157–61.

93. Sharma A, Malhotra S, Grover S, Jindal SK. Incidence, prevalence, risk factor and outcome of delirium in intensive care unit: a study from India. Gen Hosp Psychiatry. 2012;34(6):639–46.

94. Shaughnessy L. Introducing delirium screening in a cardiothoracic critical care unit. Nurs Crit Care. 2013;18(1):8–13.

95. Simeone S, Pucciarelli G, Perrone M, Teresa R, Gargiulo G, Guillari A, et al. Delirium in ICU patients following cardiac surgery: An observational study. J Clin Nurs (John Wiley Sons, Inc). 2018;27(9–10):1994–2002.

96. Simons KS, van den Boogaard M, Hendriksen E, Gerretsen J, van der Hoeven JG, Pickkers P, et al. Temporal biomarker profiles and their association with ICU acquired delirium: a cohort study. Crit Care. 2018;22(1):137.

97. Smit L, Dijkstra-Kersten SMA, Zaal IJ, van der Jagt M, Slooter AJC. Haloperidol, clonidine and resolution of delirium in critically ill patients: a prospective cohort study. Intensive Care Med. 2021;47(3):316–24.

98. Smulter N, Lingehall HC, Gustafson Y, Olofsson B, Engström KG. Delirium after cardiac surgery: Incidence and risk factors. Interact Cardiovasc Thorac Surg. 2013;17(5):790–6.

99. Soehle M, Dittmann A, Ellerkmann RK, Baumgarten G, Putensen C, Guenther U. Intraoperative burst suppression is associated with postoperative delirium following cardiac surgery: A prospective, observational study. BMC Anesthesiol. 2015;15:61.

100. Stransky M, Schmidt C, Ganslmeier P, Grossmann E, Haneya A, Moritz S, et al. Hypoactive delirium after cardiac surgery as an independent risk factor for prolonged mechanical ventilation. J Cardiothorac Vasc Anesth. 2011;25(6):968–74.

101. Su X, Meng ZT, Wu XH, Cui F, Li HL, Wang DX, et al. Dexmedetomidine for prevention of delirium in elderly patients after non-cardiac surgery: a randomised, double-blind, placebo-controlled trial. Lancet. 2016;388(10054):1893–902.

102. Sun L, Jia P, Zhang J, Zhang X, Zhang Y, Jiang H, et al. Production of inflammatory cytokines, cortisol, and Aβ1-40 in elderly oral cancer patients with postoperative delirium. Neuropsychiatr Dis Treat. 2016;12:2789–95.

103. Svenningsen H, Egerod I, Videbech P, Christensen D, Frydenberg M, Tonnesen EK. Fluctuations in sedation levels may contribute to delirium in ICU patients. Acta Anaesthesiol Scand. 2013;57(3):288–93.

104. Svenningsen H, Tønnesen E. Delirium incidents in three Danish intensive care units. Nurs Crit Care. 2011;16(4):186–92.

105. Szwed K, Pawliszak W, Szwed M, Tomaszewska M, Anisimowicz L, Borkowska A. Reducing delirium and cognitive dysfunction after off-pump coronary bypass: A randomized trial. J Thorac Cardiovasc Surg. 2021;161(4):1275–82.

106. Tate JA, Sereika S, Divirgilio D, Nilsen M, Demerci J, Campbell G, et al. Symptom communication during critical illness: The impact of age, delirium, and delirium presentation. J Gerontol Nurs. 2013;39(8):28–38.

107. Thom RP, Bui MP, Rosner B, Teslyar P, Levy-Carrick NC, Klompas M. A Comparison of Early, Late, and No Treatment of Intensive Care Unit Delirium With Antipsychotics: A Retrospective Cohort Study. Prim care companion CNS Disord. 2018;20(6):18m02320.

108. Tilouche N, Hassen MF, Ali HBS, Jaoued O, Gharbi R, El Atrous S. Delirium in the Intensive Care Unit: Incidence, risk factors, and impact on outcome. Indian J Crit Care Med. 2018;22(3):144–9.

109. Tobar E, Romero C, Galleguillos T, Fuentes P, Cornejo R, Lira MT, et al. Confusion assessment method for diagnosing delirium in ICU patients (CAM-ICU): Cultural adaptation and validation of the Spanish version. Med Intensiva. 2010;34(1):4–13.

110. Torres-Contreras CC, Paez-Esteban AN, Hinestrosa-Diaz Del Castillo A, Rincon-Romero MK, Amaris-Vega A, Martinez-Patino JP. Factors associated with delirium in critical patients in a health institution in Bucaramanga, Colombia. Enferm Intensiva. 2018/06/18. 2019;30(1):13–20.

111. Tsuruta R, Nakahara T, Miyauchi T, Kutsuna S, Ogino Y, Yamamoto T, et al. Prevalence and associated factors for delirium in critically ill patients at a Japanese intensive care unit. Gen Hosp Psychiatry. 2010;32(6):607–11.

112. Uguz F, Kayrak M, Çíçek E, Kayhan F, Ari H, Altunbas G. Delirium following acute myocardial infarction: incidence, clinical profiles, and predictors. Perspect Psychiatr Care. 2010;46(2):135–42.

113. van Dellen E, van der Kooi AW, Numan T, Koek HL, Klijn FAM, Buijsrogge MP, et al. Decreased functional connectivity and disturbed directionality of information flow in the electroencephalography of intensive care unit patients with delirium after cardiac surgery. Anesthesiology. 2014;121(2):328–35.

114. Van Den Boogaard M, Schoonhoven L, van Achterberg T, van der Hoeven JG, Pickkers P. Haloperidol prophylaxis in critically ill patients with a high risk for delirium. Crit Care. 2013;17(1):R9.

115. van den Boogaard M, Schoonhoven L, van der Hoeven JG, van Achterberg T, Pickkers P. Incidence and short-term consequences of delirium in critically ill patients: A prospective observational cohort study. Int J Nurs Stud. 2012;49(7):775–83.

116. Van Der Kooi AW, Zaal IJ, Klijn FA, Koek HL, Meijer RC, Leijten FS, et al. Delirium detection using EEG: What and how to measure. Chest. 2015;147(1):94–101.

117. van Eijk MM, van den Boogaard M, van Marum RJ, Benner P, Eikelenboom P, Honing ML, et al. Routine use of the confusion assessment method for the intensive care unit: a multicenter study. Am J Respir Crit Care Med. 2011;184(3):340–4.

118. van Eijk MM, van Marum RJ, Klijn IA, de Wit N, Kesecioglu J, Slooter AJ. Comparison of delirium assessment tools in a mixed intensive care unit. Crit Care Med. 2009;37(6):1881–5.

119. Van Ewijk CE, Jacobs GE, Girbes ARJ. Unsuspected serotonin toxicity in the ICU. Ann Intensive Care. 2016;6:85.

120. van Keulen K, Knol W, Belitser S V, Zaal IJ, van der Linden PD, Heerdink ER, et al. Glucose variability during delirium in diabetic and non-diabetic intensive care unit patients: A prospective cohort study. PLoS One. 2018;13(11):e0205637.

121. Voils SA, Shoulders BR, Singh S, Solberg LM, Garrett TJ, Frye RF. Intensive Care Unit Delirium in Surgical Patients is Associated with Upregulation in Tryptophan Metabolism. Pharmacotherapy. 2020/04/05. 2020;40(6):500–6.

122. Vyveganathan, L.; Izaham, A.; Wan Mat, W. R.; Peng, S. T. S.; Rahman, R. A.; Manap NA. Delirium in critically ill patients: Incidence, risk factors and outcomes. Crit Care Shock. 2019;22(1):25–40.

123. Wang C, Wu Y, Yue P, Ely EW, Huang J, Yang X, et al. Delirium assessment using Confusion Assessment Method for the Intensive Care Unit in Chinese critically ill patients. J Crit Care. 2013;28(3):223–9.

124. Wang J, Ji Y, Wang N, Chen W, Bao Y, Qin Q, et al. Establishment and validation of a delirium prediction model for neurosurgery patients in intensive care. Int J Nurs Pract. 2020;26(4):e12818.

125. Wang J, Ji Y, Wang N, Chen W, Bao Y, Qin Q, et al. Risk factors for the incidence of delirium in cerebrovascular patients in a Neurosurgery Intensive Care Unit: A prospective study. J Clin Nurs (John Wiley Sons, Inc). 2018;27(1–2):407–15.

126. Whitlock EL, Torres BA, Lin N, Helsten DL, Nadelson MR, Mashour GA, et al. Postoperative delirium in a substudy of cardiothoracic surgical patients in the BAG-RECALL clinical trial. Anesth Analg. 2014;118(4):809–17.

127. Wøien H, Balsliemke S, Stubhaug A. The incidence of delirium in Norwegian intensive care units; deep sedation makes assessment difficult. Acta Anaesthesiol Scand. 2013;57(3):294–302.

128. Yaşayacak A, Eker F. Determining delirium and risk factors of patients in cardiovascular surgery intensive care unit. Turkish J Thorac Cardiovasc Surg. 2012;20:265–74.

129. Zhang W, Sun Y, Liu Y, Qiu W, Ye X, Zhang G, et al. A nursing protocol targeting risk factors for reducing postoperative delirium in patients following coronary artery bypass grafting: Results of a prospective before-after study. Int J Nurs Sci. 2017;4(2):81–7.

130. Zhang W, Hu W, Shen M, Ye X, Huang Y, Sun Y. Profiles of delirium and the clinical outcomes of patients who underwent coronary artery bypass grafting: a prospective study from China. J Clin Nurs (John Wiley Sons, Inc). 2016;25(5–6):631–41.

131. Zujalovic B, Mayer B, Hafner S, Balling F, Barth E. AChE-activity in critically ill patients with suspected septic encephalopathy: a prospective, single-centre study. BMC Anesthesiol. 2020;20(1):287.

# List of extracted data

The following study characteristics were extracted:

- Author
- Publication year
- Country
- Study design
- Number of participating centers
- Type of ICU(s)
- Inclusion/exclusion criteria
- Sample size
- Delirium assessment tool(s)
- Delirium assessor(s)
- Frequency of delirium screening
- Delirium motor subtype definition

The following data were extracted for the whole study population:

- Baseline demographics (age, sex, disease severity score (SAPSII, SAPSIII, APACHEII, SOFA))
- Proportion of mechanically ventilated patients
- Duration of mechanical ventilation
- Prevalence/incidence of delirium
- ICU & hospital length of stay
- ICU & hospital mortality rates

The following data were extracted for the delirious population:

- Baseline demographics (age, sex, severity score (SAPSII, SAPSIII, APACHEII, SOFA))
- Distribution of delirium motor subtypes
- Delirium duration
- ICU & hospital length of stay
- ICU & hospital mortality rates

The following data were extracted for each delirium motor subtype:

- Delirium duration
- ICU & hospital length of stay
- ICU & hospital mortality rates
- Delirium targeted pharmacological strategy during ICU stay
- Administration of specific agents (as listed beneath) independent of indication during ICU stay:
  - Antipsychotics
  - Alfa2-agonists
  - Benzodiazepines
  - Propofol

#

# Forest plots

Figure S1 Delirium incidence

**
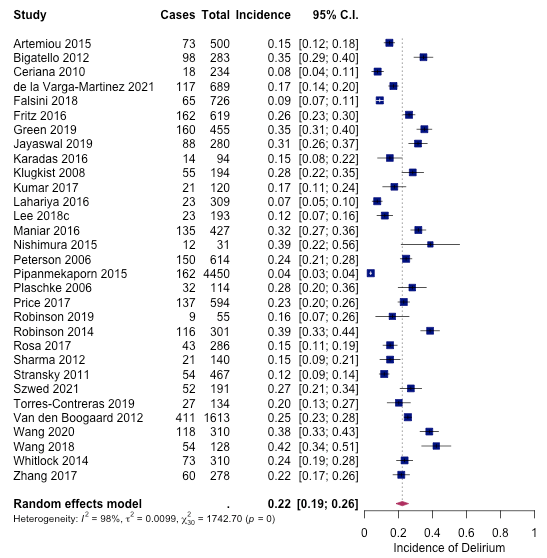
**

Figure S2 Delirium prevalence


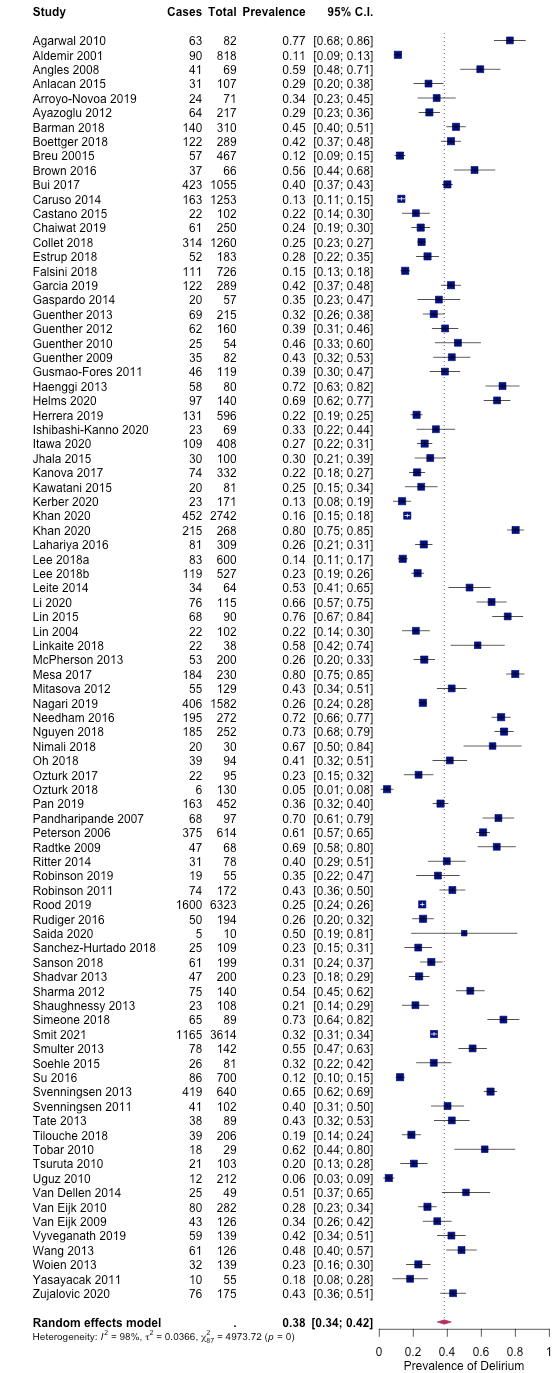


Figure S3 Distribution of hypoactive delirium


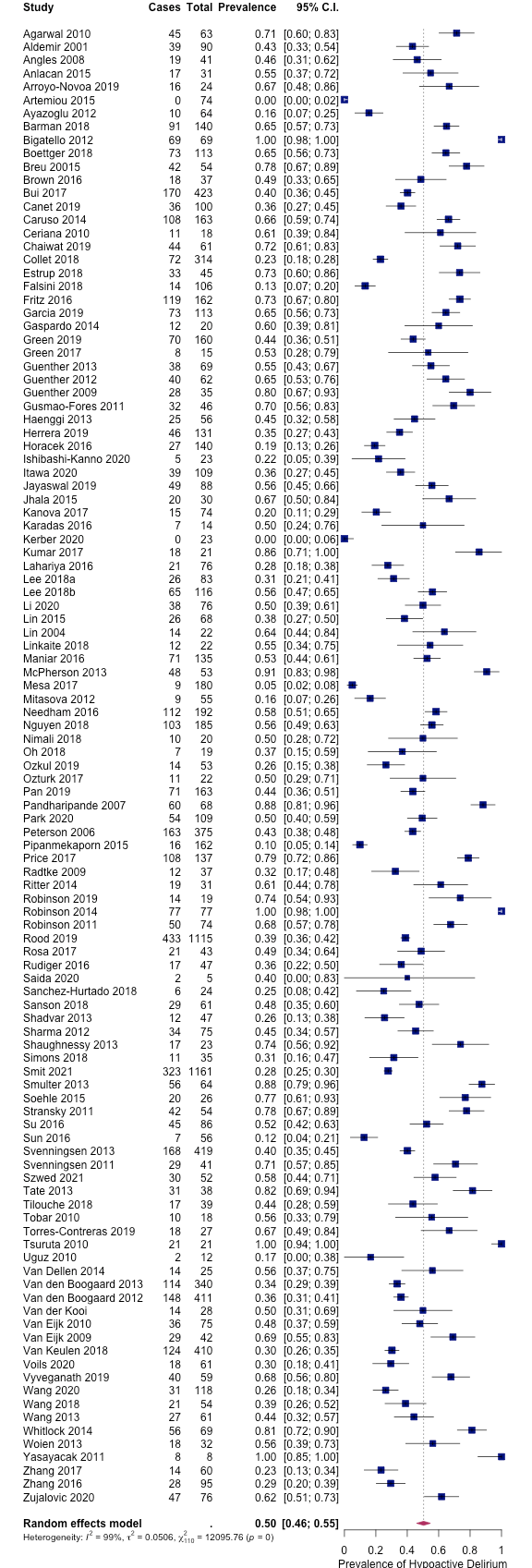


Figure S4 Distribution of hyperactive delirium


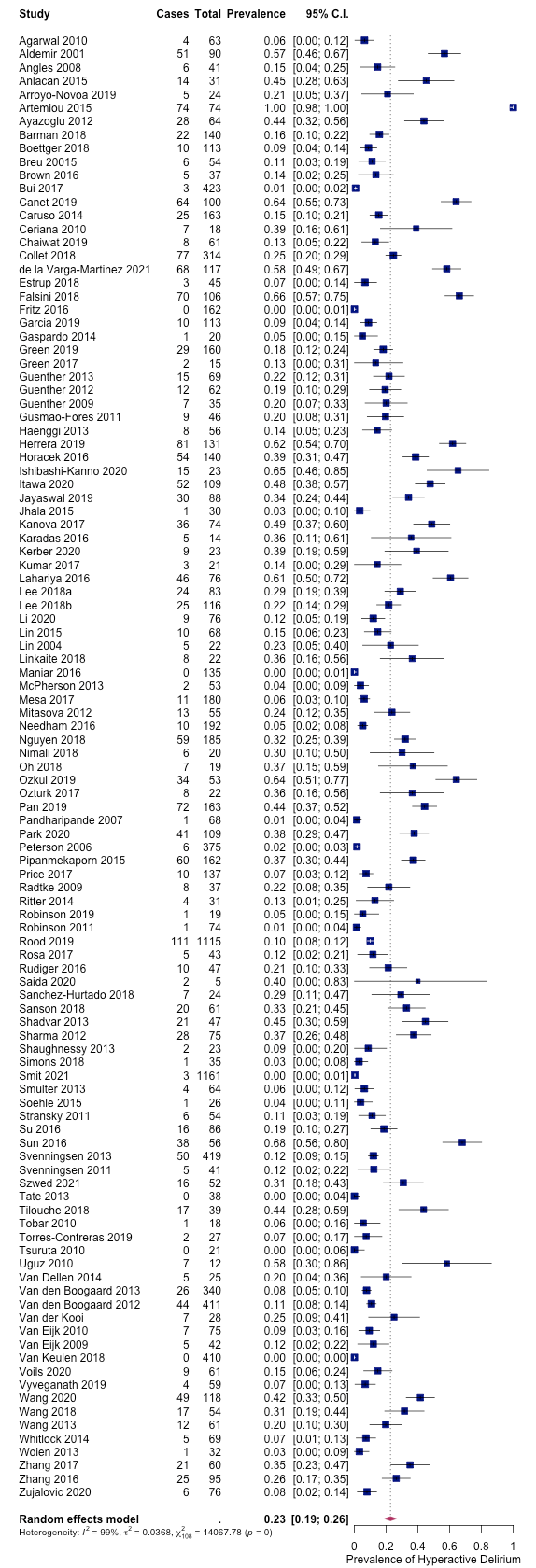


Figure S5 Distribution of mixed delirium


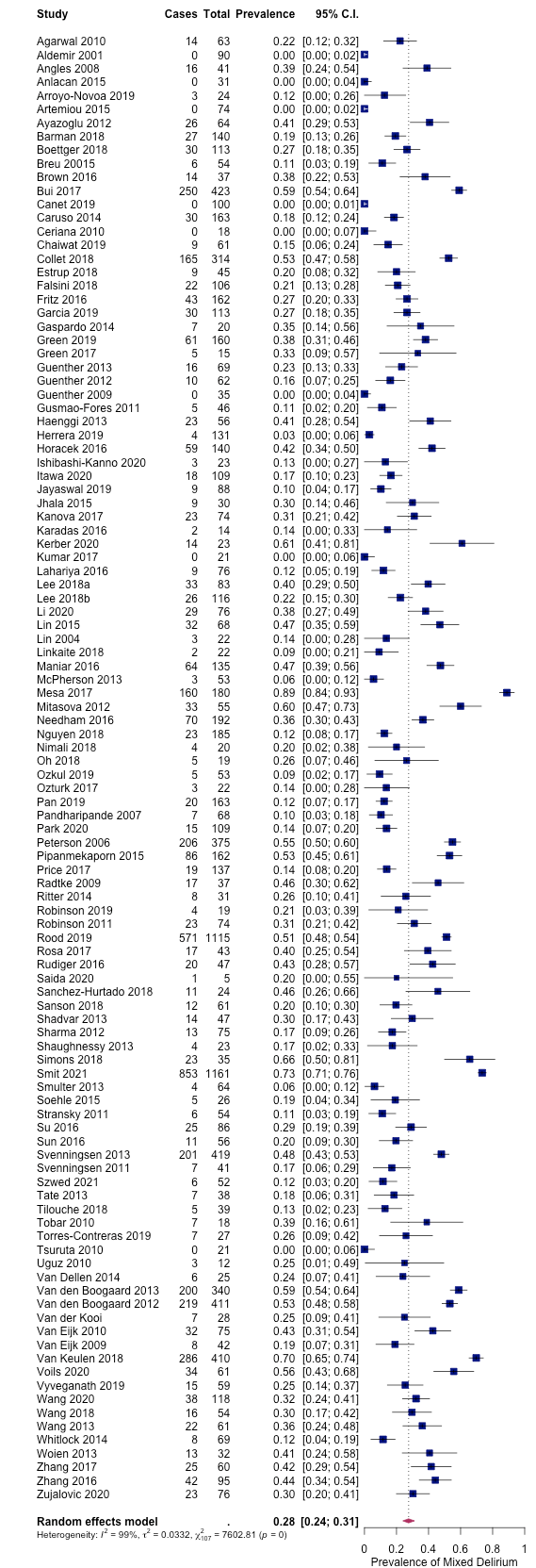


# Delirium Assessment Methods

Table S2 Delirium assessment methods

| **Delirium assessment** | **No. of studies, n (%)** |
| --- | --- |
| Delirium assessment method*  CAM-ICU  ICDSC  DSM III-V  Other | 114 (87.0)  17 (12.9)  24 (18.3)  14 (10.7) |
| Motor subtype method*  RASS  Other  N/A | 113 (86.3)  18 (13.7)  6 (4.6) |
| Assessor*  Nurse  Physician/intensivist  Psychiatrist  Research staff  Other  N/A | 68 (51.9)  33 (25.2)  19 (14.5)  30 (22.9)  2 (1.5)  12 (9.2) |
| No. of delirium assessments pr. day  ≤1  >1 ≤ 2  >2  N/A | 51 (38.9)  37 (28.2)  20 (15.3)  21 (16.0) |
| Delirium assessment period  Throughout ICU stay  1-5 days  6-10 days  >10 days  N/A | 75 (57.3)  20 (15.3)  13 (9.9)  7 (5.3)  15 (11.5) |

CAM-ICU=Confusion Assessment Method for the ICU, ICDSC = Intensive Care Delirium Screening Checklist, DSM III-V = Diagnostic and Statistical Manual of Mental Disorders III-V, RASS = Richmond Agitation and Sedation Scale, SAS = Riker Sedation-Agitation Scale

*Studies may apply more than one delirium assessment method / delirium assessor

# Characteristics of total cohort

Table S3 Characteristics of total cohort

| Characteristics of all patients (n=50,232) | Studies reporting on outcome (n) | Patients in studies reporting on outcome (n) | Pooled mean or  pooled proportion | 95% CI |
| --- | --- | --- | --- | --- |
| Age | 124 | 48,686 | 63.3 years | 62.0 – 64.5 |
| Sex (Male) | 125 | 48,193 | 61.7% | 59.7 - 63.7 |
| Severity of disease, score  APACHEII  SAPSII  SAPSIII  SOFA | 58  13  2  21 | 18,048  3,770  1,542  9,158 | 17.2  38,9  43.2  6.7 | 15.7 – 18.7  30.3 – 47.4  30.5 – 55.8  5.3 – 8.1 |
| MV in the ICU  Proportion  Duration | 88  41 | 39,278  22,320 | 64.4%  3.6 days | 57.6 – 71.1  2.8 – 4.5 |
| Length of stay  ICU  Hospital | 79  54 | 39,394  32,743 | 6.1 days  14.4 days | 5.4 – 6.9  13.0 – 15.7 |
| Mortality  ICU  Hospital | 33  27 | 13,381  9,143 | 11.4%  12.8% | 8.4 – 14.5  8.0 – 17.7 |
| Delirium occurrence  Prevalence*  Incidence** | 88  31 | 34,064  15,139 | 38.3%  22.2% | 34.2 – 42.4  18.6 – 25.8 |

APACHE = Acute Physiology and Chronic Health Evaluation, SAPS = Simplified Acute Physiology Score, SOFA = Sequential Organ Failure Assessment, MV = Mechanical ventilation

*Delirium occurrence in the ICU including delirium cases which could have occurred before ICU admission

**Newly developed delirium in the ICU
